# Risk of intestinal cancer in Crohn’s disease: re-analysis and meta-regression of population-based cohort studies

**DOI:** 10.1093/ecco-jcc/jjag013

**Published:** 2026-02-28

**Authors:** Enrico Altiero Giusto, Paolo Pinton, Carlotta Giorgi, Grazia Serino, Gianluigi Giannelli, Rossella Donghia, Francesco Fiorica

**Affiliations:** Department of Medical Sciences, Section of Experimental Medicine and Laboratory of Technologies for Advanced Therapy (LTTA), University of Ferrara, Ferrara, Italy; Azienda Ospedaliero-Universitaria S. Anna, Ferrara, Italy; Department of Medical Sciences, Section of Experimental Medicine and Laboratory of Technologies for Advanced Therapy (LTTA), University of Ferrara, Ferrara, Italy; Maria Cecilia Hospital, GVM Care & Research, Cotignola, Italy; Department of Medical Sciences, Section of Experimental Medicine and Laboratory of Technologies for Advanced Therapy (LTTA), University of Ferrara, Ferrara, Italy; Biomedical Research Center, Kansai Medical University, Osaka, Japan; National Institute of Gastroenterology—IRCCS “Saverio de Bellis,” Castellana Grotte, Italy; National Institute of Gastroenterology—IRCCS “Saverio de Bellis,” Castellana Grotte, Italy; National Institute of Gastroenterology—IRCCS “Saverio de Bellis,” Castellana Grotte, Italy; Department of Clinical Oncology, Section of Radiation Oncology and Nuclear Medicine, Verona, Italy; Department of Clinical Oncology, Section of Medical Oncology, Verona, Italy

**Keywords:** neoplasms, Crohn's disease, intestine, inflammatory bowel disease

## Abstract

**Background and aims:**

The risk of intestinal malignancy in Crohn’s disease (CD) varies across regions. Given the rising global cancer prevalence, this study aimed to summarize evidence and evaluate associations between CD and site-specific intestinal cancer risks.

**Methods:**

We systematically searched PubMed, Embase, and Scopus from inception to July 10, 2025. All population-based epidemiological observational studies that investigated any outcome related to intestinal cancer in adults (the majority of participants >18 years of age), including individuals of any ethnicity or sex from all countries and settings that report observed and expected national cases, were included. Patients diagnosed with colorectal cancer (CRC) or small bowel cancer (SBC) less than 1 year after CD diagnosis, as well as papers that address cancer mortality while combining CD and ulcerative colitis outcomes, referral-center studies, and review articles, were excluded. We analyzed 27 population-based studies using restricted maximum-likelihood models; pooled standardized incidence ratios (SIRs) were calculated for CRC and SBC. Meta-regression explored the impact of cohort size, follow-up duration, calendar year, and disease location on risk estimates. Methodological quality was assessed with the modified Newcastle–Ottawa Scale.

**Results:**

The overall CRC SIR in CD patients was significantly elevated (SIR 2.20, 95% CI 1.69-2.86). Colon cancer (SIR 2.06, 95% CI 1.09-3.90) and rectal cancer (SIR 1.75, 95% CI 1.16-2.62) risks were higher, especially in colonic disease (SIR 3.29, 95% CI 1.66-6.50) and ileocolonic disease (SIR 4.13, 95% CI 2.14-7.94), while ileal disease exposed a milder risk (SIR 1.95, 95% CI 1.16-3.26). SBC SIR was markedly increased (SIR 17.18, 95% CI 9.93-29.73), particularly in ileal (SIR 44.85, 95% CI 12.93-155.54) and ileocolonic disease (SIR 21.44, 95% CI 5.13-89.62).

**Conclusion:**

CD is associated with heightened CRC and SBC risks, varying by disease location. These findings underscore the need for tailored cancer screening and further research into the impact of environmental and genetic factors on cancer risk in CD patients.

## Graphical abstract

**Graphical Abstract:**
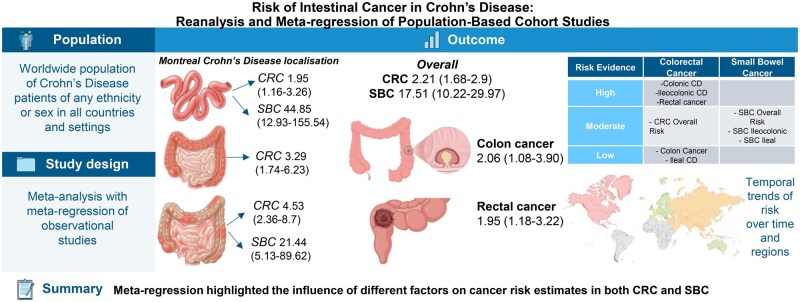

## 1. Introduction

The risk of intestinal malignancy in Crohn’s disease (CD) remains uncertain, despite extensive research into colorectal cancer (CRC) in ulcerative colitis (UC).^1^^,^^2^ While several studies have suggested an increased risk of CRC among CD patients,^3^^,^^4^ others have found no such association.^5–7^ Similarly, estimates of small bowel cancer (SBC) risk in CD vary widely.^8^

The global incidence of CD has been rising steadily: over the last decades, the incidence has continued to rise worldwide, reaching rates of 16.6/100 000 in North America and 9.8/100 000 in Europe.^9^

Among these risk factors, disease duration emerges as a significant predictive factor, while the presence of concomitant primary sclerosing cholangitis represents an additional risk, suggesting a potential association between these two inflammatory conditions and the predisposition to intestinal neoplasms. Given the increased mortality associated with a neoplastic condition compared to CD alone, timely preventive and diagnostic strategies become even more crucial. Consequently, it is hypothesized that controlling inflammation may reduce the risk of intestinal neoplasia. This hypothesis underlies many therapeutic strategies in CD; however, definitive evidence of a reduced oncological risk solely through inflammation control requires further long-term prospective studies.^10^

This rate increases the strain on healthcare systems. A better understanding of disease prognosis is essential to optimize patient care, manage the growing patient population, and prevent patients from facing unwarranted restrictions in life insurance.

This study aimed to conduct a meta-analysis to assess the overall risk of CRC and small bowel cancer in CD patients. The analysis was derived from population-based cohorts and compared the cancer risks to expected rates in age- and gender-matched background populations. Additionally, the study sought to determine whether factors such as cohort size, publication year, calendar period of observation, location of CD, or time of follow-up influenced the observed risk of intestinal cancer. Despite several recent systematic reviews and meta-analyses investigating CRC or SBC in CD patients, there has yet to be a comprehensive synthesis that jointly evaluates the risks of both cancers in population-based studies while also exploring how study-level characteristics influence reported cancer risks. We therefore conducted a meta-analysis with meta-regressions to quantify the risk of CRC and SBC among patients with CD, compared to background populations. Additionally, we aimed to examine the influence of factors such as cohort size, calendar period, publication year, cancer site (L1 for ileal, L2 for colonic, and L3 for ileocolonic), and duration of follow-up on the reported estimates. To date, the majority of other systematic reviews and meta-analyses have focused solely on odds ratios, relative risks, and hazard ratios. Moreover, those that attempted a comprehensive epidemiological analysis lacked sufficient data.

## 2. Methods

Our study protocol was registered with PROSPERO (registration number: CRD42024609923). We followed a standardized methodology^11^ and report findings according to the Preferred Reporting Items for Systematic Reviews (ie, PRISMA^12^) (Table S1) and Meta-analyses of Observational Studies in Epidemiology (ie, MOOSE^13^) (Table S2) recommendations.

### 2.1. Inclusion/exclusion criteria

The full texts of potentially eligible articles were independently reviewed by two authors, E.A.G. and F.F. Conflicts were resolved by consensus with a third author, C.G.

We focused on articles that reported epidemiological studies examining any intestinal cancer outcomes in adults, defined as individuals primarily over 18 years of age, regardless of ethnicity or sex, and conducted in any country and setting. We searched for observational cohort studies that were population-based, ensuring that the observed and expected cases were matched with the background population reported by each country.

This meta-analysis incorporates studies that examined population-based cohorts of CD patients diagnosed according to well-defined criteria; primary studies used the International Classification of Disease (ICD) codes to assess the diagnosis of CD.

To be included, studies were required to provide comprehensive data on the total number of patients, follow-up duration, exact counts of colorectal and/or small bowel cancers, and/or rates of observed-to-expected cancers matched to the background population, with confidence intervals (CIs) at 95% or enough data to calculate them.

Patients with CRC or SBC less than 1 year after CD diagnosis were excluded.

**Figure 1.**
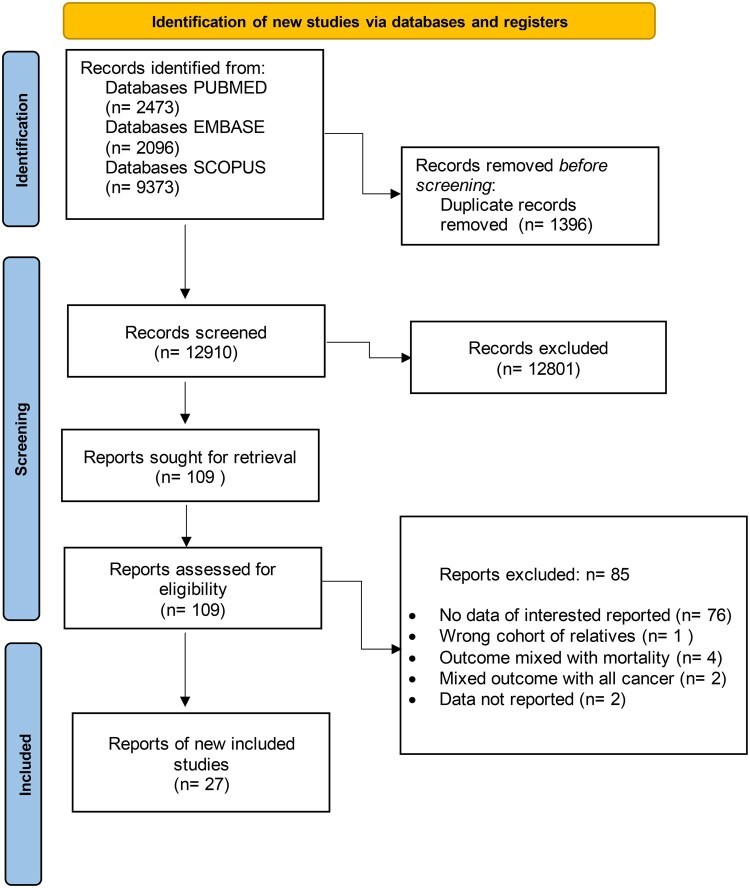
PRISMA summary of the evidence search and selection process.

Papers focused on cancer mortality, those combining CD and ulcerative colitis outcomes, referral-center studies, and review articles were excluded. In instances of duplicate publications, the most recent study with the longest follow-up of patients was considered (Figure 1).

The present systematic review was conducted and reported in accordance with the PRISMA 2020 guidelines, as detailed in Table S1. All key reporting items were addressed, including the title and abstract, rationale and objectives, eligibility criteria, search strategy, study selection and data collection, risk of bias assessment, synthesis methods (including meta-analysis, assessment of heterogeneity, subgroup, and sensitivity analyses), reporting biases, and certainty of the evidence. Results are presented using structured tables and figures, while the discussion provides an interpretation of the findings, addresses study limitations, and outlines implications for clinical practice and future research. Information regarding registration, protocol, funding, competing interests, and data availability is transparently reported.

In addition, the reporting of observational studies followed the MOOSE guidelines, as outlined in Table S2. All major recommended items were considered, including background and problem definition, hypothesis formulation, study outcomes and exposures, study design and population, comprehensive search strategy, and methodological aspects such as study selection and coding, assessment of confounding and study quality, evaluation of heterogeneity, and statistical methods. In line with these recommendations, potential sources of bias were carefully considered when interpreting the results. In particular, the elevated standardized incidence ratios for cancer risk in patients with CD may be influenced by several factors. These include misclassification of disease location due to changes in diagnostic coding practices over time, immortal time bias arising from the exclusion of cancers diagnosed shortly after the CD diagnosis, and left censoring related to the exclusion of cancers present prior to cohort entry. All of these factors may contribute to an overestimation of risk. Moreover, differential surveillance bias cannot be excluded, as patients with CD undergo more intensive clinical monitoring than the general population, potentially increasing cancer detection rates and consequently standardized incidence ratio estimates.

### 2.2. Search strategy

To identify all relevant papers in English that address the risk of intestinal cancer in patients with CD, based on well-defined population-based cohorts, we conducted a systematic and exhaustive search using PubMed, Embase, and Scopus as search engines from January 1, 1966, until July 10, 2025. We used MESH terms, truncated words, and exploded terms: CD epidemiology terms combined with a free text search on the words: “cancer” OR “neoplasm” OR “neoplasia” OR “tumor” OR “adenocarcinoma” OR “carcinoma” with the following filter: studies of humans (for the complete search strategy, see the supplementary material) to include all papers. In addition, reference lists of all included papers and their related articles were scrutinized in search of additional literature on the topic.

### 2.3. Data extraction

Data extraction was performed independently by two authors (E.A.G. and F.F.). Disagreements were resolved by consensus with P.P. The included papers were reviewed in detail for data on the number of patients studied, calendar year of publication, calendar period of inclusion and observation, mean or median duration of follow-up, prevalence of ileal, ileocolonic, and colonic disease, number of small bowel, colon, and rectum cancers observed in the cohort, expected numbers in a matched background population, and/or observed to expected cancer rates with 95% CI. We used the Montreal classification to define the extent of the disease in primary studies.

### 2.4. Strength of epidemiological evidence

To align our approach with the GRADE framework, we considered additional domains for each outcome: risk of bias (study quality), inconsistency (*I*^2^ and direction of effect), indirectness (population, intervention, comparator, or outcome differences), imprecision (width of CIs), and publication bias (funnel plot asymmetry). Outcomes were downgraded or upgraded based on these criteria. For example, an outcome with a low *I*^2^ but a high risk of bias was downgraded to “moderate,” while consistent effects across multiple high-quality studies were upgraded to “high.” This approach ensures that the level of evidence for each outcome reflects both statistical and methodological considerations. *I*^2^ levels (%), which were considered based on their values, were classified as follows: <40%, low; 30-60%, moderate; 50-90%, substantial; 75-100%, considerable.

### 2.5. Quality assessment of the meta-analysis methodology

We assessed the methodological quality of the studies by means of the modified Newcastle–Ottawa Scale (Modified NOS),^12^ a strict, validated, and reliable measurement tool that is designed to evaluate cohort studies. It consists of eight items and includes ratings for selection, comparability, outcome, and follow-up adequacy.^13^ It results in an assessment of the methodological quality in one of three grades: high, moderate, or low (Table S3).

### 2.6. Data analysis

Meta-analysis used restricted maximum likelihood (REML)^14^^,^^15^ for estimating variance components in mixed-effects models to estimate the between-study variance. The influence of the calendar year of publication, cohort size, observation time, or percentage of the population affected by colon or small bowel disease on the standardized incidence ratio (SIR) of CRC and SBC, respectively, was tested by meta-regression analyses with the REML method. Sensitivity analyses were performed on meta-regressions.

The concept of heterogeneity and methods to assess them are discussed in the supplementary material.

Subsequently, pooled SIRs and meta-analysis were calculated using Stata19 (StataCorp., College Station, TX, USA) and R (R Foundation for Statistical Computing, Vienna, Austria, 2024; https://www.R-project.org).

We presented the extracted estimates of random effects by means of forest plots displaying the association between each factor and CD. According to the heterogeneity test (significance at a 5% level), either a fixed or a random effects model was applied.

If SIRs (observed/expected numbers) or identical estimates for CRC and SBC were reported without 95% CI, the interval was calculated using the observed and expected number of cancers and assuming a Poisson distribution of observed cases utilizing the Garwood method (see supplementary material).

The observed cases are the cancer cases seen in the study population, while the expected cases in primary studies were obtained from national cancer institutes such as SEER (USA), NORDCAN (Nordic countries), GLOBOCAN or CI5 from IARC/WHO, and national registries (eg, AIRTUM in Italy and NHS Digital in the UK). The expected number of cases is estimated by applying age-, sex-, and calendar year-specific cancer incidence rates from the general population to the person-year at risk in the study population, usually 100 000 persons, multiplying the person-year (total follow-up for each stratum) by the cancer incidence rate in the general population for that stratum, and then summing all strata.

Subgroup analyses were performed for both CRC and SBC: pre- and post-1997 (beginning of the biologic era), categorized by ICD for the middle year and ICD for the beginning of the study; furthermore, to avoid possible cohort overlapping or double counting, a study for each nation was selected, and subsequent analyses for longest follow-up, major number of observed cancer cases, and largest population were made.

### 2.7. Ethics statement

This meta-analysis was conducted using data extracted from previously published population-based cohort studies. All included studies had obtained the necessary ethical approvals from their respective institutional review boards, as documented in the original publications.

## 3. Results

### 3.1. Quality assessment of meta-analyses

The Modified NOS rating for all studies was determined to be high (27 studies, 100.00%), with two studies awarded 9 stars (8.00%). The most common flaws were limited patients with a CD population under 1000 patients (*n* = 10, 34.00%), disease extent or cancer location (*n* = 13, 50.00%), and follow-up under 10 years (*n* = 11, 42.00%).

### 3.2. Colorectal cancer risk

Twenty-three studies^3^^,^^5^^,^^7^^,^^8^^,^^16–33^ were eligible for our study, reporting exact numbers of observed or expected CRCs and/or ratios of observed to expected numbers or enough data to calculate them, with SIRs ranging from 0.81 (95% CI 0.39-1.50) to 6.9 (95% CI 2.8-14.2) (Table 1). The mean follow-up time of patients was 11.2 years with 340 observed cancer cases.

**Table 1.** Summary of papers included in the meta-analysis.

Author, Country	Calendar period (Publication year)	No. of patients	Mean or median follow-up (years)	Observed/expected no. of CRCs	SIR of CRC (95% CI)	Observed/expected no. of SBCs	SIR of SBC (95% CI)
**Axelrad, Denmark-Sweden**	1969-2017 (2020)	4737	11.60	–	–	154/13.94	11.04 (9.15-12.93)
**Beaugerie, France**	2004-2007 (2013)	11 759	2.92	19/7.77	2.44 (1.47-3.82)	–	–
**Bernstein, Canada**	1984-1987 (2001)	2857	7.47	29/-	2.64 (1.69-4.12)	7/0.4	17.4 (4.16-72.9)
**Bojensen, Denmark**	1978-2010 (2017)	20 917	11.55	–	–	32/2.92	10.96 (7.76-15.51)
**Cheddani, France**	1988-2006 (2009)	370	6.0	7/5.8	1.2 (1.16-8.23)	–	–
**Ekbom, Sweden**	1965-1983 (1990)	1469	11.0	12/4.9	2.2 (1-4.3)	1/0.3	3.3 (0.1-18.6)
**Fireman, Israel**	1970-1980 (1989)	274	10.0	1/0.88	1.14 (0.03-6.33)	–	–
**Gatenby, New Zealand**	2003-2020 (2021)	649	20.4	13/3.17	4.1 (2.4-7.1)	–	–
**Greenstein, Israel**	1960-1976 (1981)	579	11.0	7/1.01	6.9 (2.8-14.2)	4/0.05	95.7 (38.5-197.3)
**Gyde, U.K.**	1944-1976 (1980))	513	14.5	9/2.07	4.34 (2-8.2)	–	–
**Jess, Denmark**	1962-1997 (2004)	374	17.0	4/3.52	1.14 (0.31-2.92)	4/0.06	66.7 (18.13-170.68)
**Jess, Denmark**	1978-2010 (2013)	313	8.0	12/6.99	0.85 (0.67-1.07)	2/0.13	–
**Jess, USA**	1940-2002 (2006)	810	14.0	6/3.2	1.87 (0.69-4.07)	3/0.07	40.6 (8.4-118)
**Jung, Korea**	2011-2014 (2017)	5506	2.14	13/3.25	4 (2.13-6.84)	4/0.09	44.4 (12.12-120.24)
**Kappelman, Denmark**	1978-2010 (2014)	13 756	32.0	69/74.6	0.9 (0.7-1.2)	12/1.4	8.4 (4.3-14.7)
**Kim, Korea**	2006-2015 (2021)	13 739	3.24	25/15.26	1.64 (1.14-2.40)	–	–
**Lee, Korea**	1989-2013 (2015)	2414	7.40	12/2	6 (3.1-10.48)	–	–
**Mellemkjaer, Denmark**	1977-1989 (2000)	2645	9.60	15/13.1	1.1 (0.6-1.9)	5/0.3	17.9 (5.8-42)
**Mizushima, Japan**	1989-2009 (2010)	294	14.45	6/1.03	5.8 (2.13-12.7)	–	–
**Persson, Swedish**	1955-1974 (1994)	1251	15.0	5/5.63	0.89 (0.29-2.07)	4/0.26	15.64 (4.26-40.06)
**Sammader, USA**	1996-2011 (2018)	5084	6.70	40/11.8	3.4 (2.3-4.4)	–	–
**So, China**	1990-2016 (2017)	1108	8.0	6/3.66	1.64 (0.74-3.65)	–	–
**Taborelli, Italy**	1995-2013 (2020)	1306	7.10	10/12.3	0.81 (0.39-1.50)	–	–
**Van den Heuvel, Holland**	1991-2011 (2016)	1157	8.10	11/5.65	1.95 (0.97-3.48)	–	–
**Yano, Japan**	1985-2010 (2013)	770	13.10	9/3.23	2.79 (1.28-5.29)	–	–
**Yu, Norway-Sweden**	1987-2016 (2022)	45 052	10.0	–	–	59/12.3	4.79 (3.72-6,19)

Abbreviations: CRC, Crohn’s disease; SBC, small bowel cancer; SIR, standardized incidence ratio.

**Figure 2.**
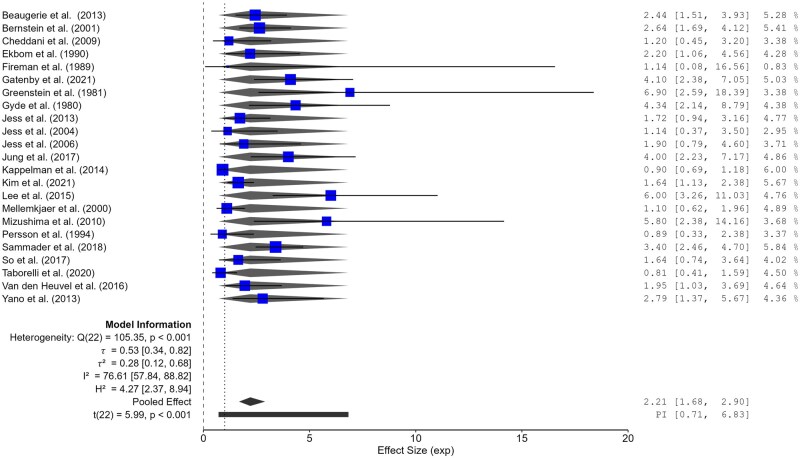
Standardized incidence ratios with 95% CI for colorectal cancer in Crohn’s disease (log scale). Individual and combined standardized incidence ratios (with 95% CI) of colorectal cancer in Crohn’s disease. The size of the boxes is proportional to the weight (1/SE) of each study.

The estimated data were pooled using the random effects model as the heterogeneity test was: τ^2 = ^0.28, *I*^2 = ^76.61%, *H*^2 = ^4.27; test of qi = qj: Q(22) = 105.35, *P* < .001. The pooled estimate revealed an increased overall SIR for CRC in CD of 2.21 (95% CI 1.68-2.90) (Figure 2).

Excluding the studies reporting the lowest and highest SIR for CRC, the result did not change: SIR = 2.01 (95% CI 1.6-2.53).

Separate risk estimates for cancer of the colon and rectum in CD patients were available from six studies (27.3%)^7^^,^^8^^,^^16^^,^^17^ with 68 and 38 observed cancers, respectively (Figures S12-S13).

**Figure 3.**
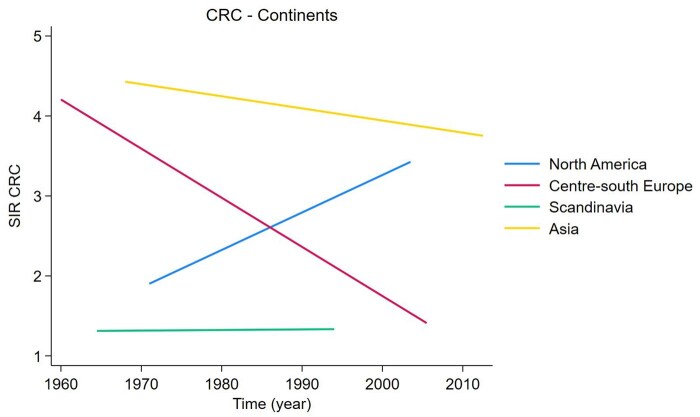
Temporal trends in colorectal cancer (CRC) plotted over continents. Each regression line corresponds to a specific continent. SIR, standardized incidence ratio.

**Figure 4.**
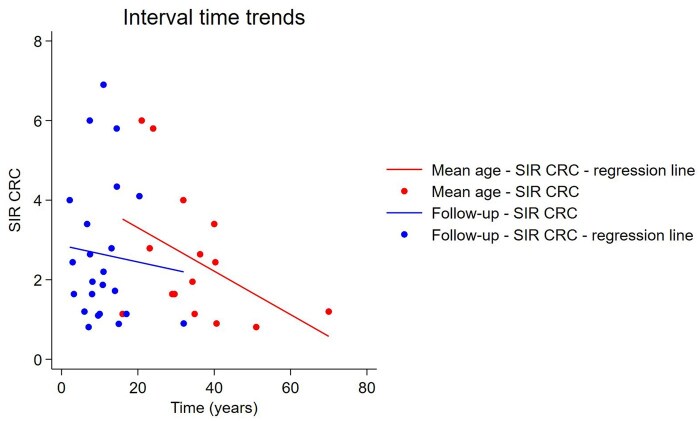
Incidence of colorectal cancer (CRC) plotted over mean age and follow-up. Each circle represents an individual study. The circle is centered around the study mean age and mean follow up on the *x*-axis plotted against the risk of CRC on the *y*-axis. Effects of time on the incidence of CRC were examined by regression techniques, and the regression lines for each geographic region are superimposed on the graph. SIR, standardized incidence ratio.

**Figure 5.**
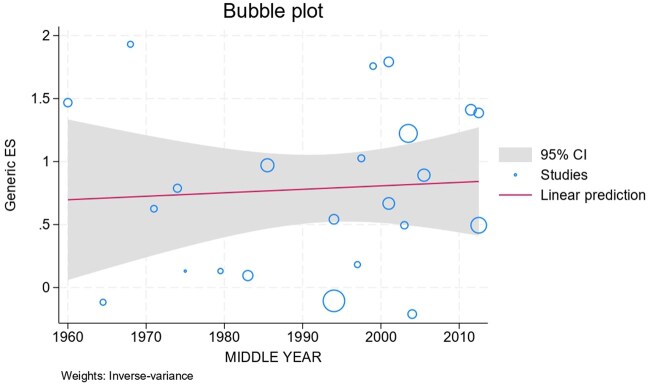
Incidence of colorectal cancer (CRC) plotted over time and regions. Each circle represents an individual study. The circle is centered around the study mid-point year on the *x*-axis plotted against the risk of CRC on the *y*-axis. The size of the circles is inversely proportional to the variance associated with the estimate of the incidence rate of CRC in each study. Effects of time on the incidence of CRC were examined by mixed meta-regression techniques, and the regression lines for each geographic region are superimposed on the graph.

We added the SIR, defined as the mean SIR for the continent (Figure 3). Additionally, the CRC SIR related to the study’s middle year and publication year was evaluated in Figure 4, and a bubble plot based on the middle year is illustrated in Figure 5.

A pre- and post-1997 analysis was conducted as shown in Figure S1. The meta-analysis included 23 studies stratified into two subgroups: POST (*n* = 14) and PRE (*n* = 9). In the POST subgroup, the pooled effect size was statistically significant, with an estimated risk of 2.54 (95% CI 1.84-3.49; *t*(13) = 6.28, *P* < .001) and a prediction interval ranging from 0.94 to 6.87. Heterogeneity was high (Q(13) = 40.23, *P* < .001), with τ = 0.44, τ^2^ = 0.19, *I*^2^ = 70.26%, and *H*^2^ = 3.36. In the PRE subgroup, the pooled effect was lower and marginally non-significant (1.73, 95% CI 0.99-3.02; *P* = .053), with a wider prediction interval (0.38-7.79). Heterogeneity remained high (Q(8) = 33.28, *P* < .001), with τ^2^ = 0.37, *I*^2^ = 76.22%, and *H*^2^ = 4.21. When considering the full dataset, the overall pooled effect was 2.21 (95% CI 1.68-2.90; *t*(22) = 5.99, *P* < .001), with a prediction 95% CI of 0.71-6.83. The model showed considerable heterogeneity (Q(22) = 105.35, *P* < .001), with τ = 0.53, τ^2^ = 0.28, and *I*^2^ = 76.61%. Moreover, analyses by ICD-middle year of primary study, ICD-start of study, follow-up, cases, and the larger population were conducted and included as Figures S2-S6. To evaluate the robustness of the meta-analytic findings, leave-one-out analyses were performed by systematically excluding each study, as shown in Figure S7.

A funnel plot illustrating publication bias is presented in Figure S8, providing a visual representation of the potential asymmetry in the study effect sizes. A Galbraith plot was used to visualize the study-specific effect sizes along with their precisions and to assess heterogeneity (see Figure S9).

The odds ratios (ORs) and 95% CIs for CRC did not change substantially when applying the “trim-and-fill method” for publication bias adjustment (see Figure S10). This suggests that publication bias is not a significant concern in this meta-analysis.

To further assess potential publication bias, we conducted the Egger regression test (see Figure S11). This Egger test showed no statistically significant evidence of publication bias (*P* = .89).

The overall pooled SIR of colon cancer was 2.06 (95% CI 1.08-3.90, *P* = 0.027, *I*^2^: 76.87%, Cochran Q test, *P* = .002; Figure S12).

On the other hand, the overall pooled SIR of rectal cancer was 1.95 (95% CI 1.18-3.22, *I*^2^: 32.13%, Cochran Q test, *P* = .23; Figure S13).

We identified a risk of CRC among these patients with SIR ileal disease, noting 14 observed cancers: 1.95 (95% CI 1.16-3.26, *P* = .012, *I*^2^: 0.00%, Cochran Q test, *P* = .57; Figure S14).

The SIRs for CRC in patients with pure colonic disease were reported in six studies with 11 cancers. We found an increased risk of CRC among these patients with SIR colonic disease: 3.29 (95% CI 1.74-6.23, *P* < .001, *I*^2^: 32.79%, Cochran Q test, *P* = .15; Figure S15).

Finally, the SIRs of CRC with ileocolonic disease, totaling 16 cases, were reported in five studies: the pooled risk was 4.53 (2.36-8.70, *P* < 0.001, *I*^2^: 32.68%, Cochran Q test, *P* = .28; Figure S16).

### 3.3. Small bowel cancer risk

Thirteen selected cohort studies^3^^,^^5^^,^^7^^,^^8^^,^^16–18^^,^^20^^,^^21^^,^^32–46^ (Table 1) provided comprehensive information on the incidence of SBC. The Fireman,^19^ Yano,^22^ and Mizushima^24^ studies had to be excluded as they only reported the observed number of SBCs (*n* = 0,^19^  *n* = 1,^22^ and *n* = 1,^24^ respectively), without providing the expected numbers.

**Figure 6.**
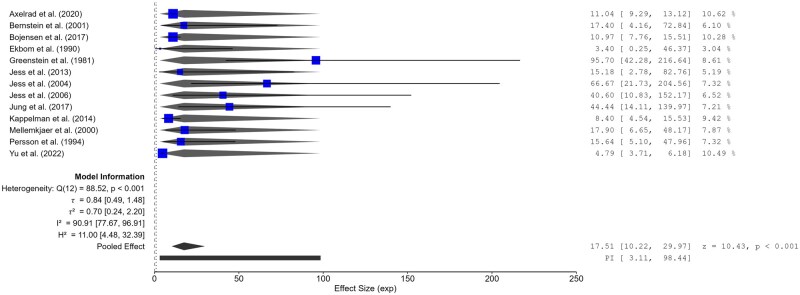
Standardized incidence ratio (SIR) of small bowel cancer (SBC) with 95% CI for colorectal cancer in Crohn’s disease (log scale). Individual and combined SIRs (with 95% CI) of colorectal cancer in Crohn’s disease. The size of the boxes is proportional to the weight (1/SE) of each study.

The pooled SIR was recalculated for the remaining 11 studies, which reported 291 observed cases of SBC. The random effects model was used as the heterogeneity test was *I*^2^ = 95.7%, Cochran Q test, *P* < .001, τ^2^ = 583. In the remaining 13 studies, SIR ranged from 3.40 (95% CI 0.25-46.37) to 95.70 (95% CI 42.28-216.64), and the overall pooled SIR of SBC was 17.51 (95% CI 10.22-29.97, *P* < .001) (Figure 6).

Exclusion of the studies with the lowest and highest SIR for SBC did not change the resulting SIR, which remained at 21.1 (95% CI 6.33-65.22).

This analysis, based on SBC studies in 1997 (Figure S17), included a total of 13 studies conducted before (PRE) and after (POST) a defined temporal threshold. In the pre-subgroup, nine studies were pooled, yielding a summary effect size of 17.85 (95% CI 9.58-33.26), with considerable heterogeneity (Q(8) = 38.03, *P* < .001; τ^2^ = 0.64; *I*^2^ = 88.32%). Individual study estimates ranged from 3.40 to 95.70. Four studies were included in the POST subgroup, resulting in a pooled effect size of 17.60 (95% CI 5.57-55.57), also with considerable heterogeneity (Q(3) = 24.85, *P* < .001; τ^2^ = 1.07; *I*^2^ = 82.26%). Across the full dataset, the overall pooled effect was 17.51 (95% CI 10.22-29.97), with pronounced heterogeneity (Q(12) = 88.52, *P* < .001; τ^2^ = 0.70; *I*^2^ = 90.91%). These findings indicate consistently elevated effect sizes across both temporal strata, albeit with considerable between-study variability.

Figure S18 displays subgroup 9, which included four studies, with predicted effect sizes ranging from 11.04 to 66.67. The pooled estimate was 19.73 (95% CI 8.96-43.44; *z* = 7.41; *P* < .001). Substantial heterogeneity was observed (Q(3) = 10.69; *P* = .014), with τ^2^ = 0.65, *I*^2^ = 70.50%, and *H*^2^ = 3.39. Subgroup 10 comprised five studies, yielding a pooled predicted effect of 12.99 (95% CI 5.76-29.30; *z* = 6.18; *P* < .001). Heterogeneity was substantial (Q(4) = 32.02; *P* < .001), with τ^2^ = 0.84, *I*^2^ = 91.72%, and *H*^2^ = 12.08. Subgroup 8 included three studies with highly variable predictions, ranging from 3.40 to 95.70. The pooled estimate was 22.96 (3.50-150.50; *z* = 3.27; *P* = 0.001), and heterogeneity was Q(2) = 88.34; *P* = 0.015, with τ^2^ = 1.42, *I*^2^ = 75.36%, and *H*^2^ = 4.06. Finally, full dataset analysis yielded a pooled predicted effect size of 17.51 (10.22 to 29.97; *z* = 10.43; *P* < .001), with strong evidence of heterogeneity across all studies (Q(12) = 88.52; *P* < 0.001), with τ^2^ = 0.84, *I*^2^ = 90.91%, and *H*^2^ = 11.00.

Additionally, as in the previous CRC section, analyses by middle year, start of study, follow-up, cases, and larger population were conducted and included as Figures S18-S22.

Leave-one-out sensitivity analyses were performed to assess the influence of individual studies on the pooled effect estimate in SBC (Figure S23).

Publication bias was evaluated using a funnel plot (see Figure S24). Study-specific effect sizes, their precision, and heterogeneity were assessed using a Galbraith plot (see Figure S25).

The trim-and-fill method was then applied to account for potential publication bias (see Figure S26). Potential publication bias was further assessed using the Egger regression test (see Figure S27), which showed no statistically significant evidence of bias (*P* = .34).

The SIR of SBC with ileal prevalence was evaluated in all five studies, with 90 observed cancers and an increased risk of SBC in these patients (SIR 44.85; 95% CI 12.93-155.54, *P* < .001, *I*^2^: 88.38%, Cochran Q test, *P* < .001) (Figure S28).

Finally, patients with ileocolonic disease were analyzed in four studies. A total of 58 instances of cancer were observed with an increased risk of SBC (SIR 21.44; 95% CI 5.13-89.62, *P* < .001, *I*^2^: 95.91%, Cochran Q test, *P* < .001) (Figure S29).

### 3.4. Meta-regression

A meta-regression analysis was performed to investigate potential sources of heterogeneity across studies on CRC and SBC (Table 2).

**Table 2.** Meta-regression analysis for colorectal cancer (CRC) and small bowel cancer (SBC) patients.

Parameters	CRC				SBC			
	β	SE (β)	*P*	95% CI	β	SE (β)	*P*	95% CI
***Univariate***								
**No. of patients**	−0.00003	0.00003	.285	−0.00009 to 0.00002	−0.00003	0.00001	.007	−0.00005 to −8.63e-06
**Males**	−0.00001	0.00006	.799	−0.0001 to 0.0001	−0.00006	0.00002	.009	−0.0001 to −0.00001
**Females**	0.00005	0.0001	.689	−0.0002 to 0.0003	−0.00005	0.0002	.019	−0.0001 to −8.88e-06
**Mean age (years)**	−0.0256	0.0130	.050	−0.0512 to 0.0001	−0.0615	0.0257	.017	−0.0112 to −0.0111
**Median follow-up (years)**	−0.0191	0.0188	.310	−0.0559 to 0.0178	−0.0237	0.0427	.580	−0.1075 to 0.0601
**Middle year**	0.0027	0.0089	.756	−0.0146 to 0.0201	−0.0491	0.0145	.001	−0.0775 to −0.0207
**Publication (year)**	−0.0071	0.0115	.540	−0.0297 to 0.0156	−0.0527	0.0132	<.001	−0.0786 to −0.0268
**Nation**	0.0009	0.0337	.979	−0.0652 to 0.0670	0.0941	0.1261	.455	−0.0153 to 0.3415
**Continent**	0.1261	0.1173	.282	−0.1039 to 0.3561	0.1362	0.3602	.705	−0.5700 to 0.8422
***Multivariate***								
**Mean age (years)**	−0.0269	0.0119	.024	−0.0503 to −0.0035	−0.0630	0.0287	.028	−0.1193 to −0.0067
**Median follow-up (year)**	−0.0321	0.0159	.044	−0.0633 to −0.0008	−0.0188	0.0281	.503	−0.0740 to 0.0363
**Middle year**	0.0520	0.0205	.011	0.0117 to 0.0923	−0.0080	0.0307	.795	−0.0681 to 0.0522
**Publication (year)**	−0.0700	0.0269	.009	−0.1228 to −0.0172	−0.0456	0.0295	.122	−0.1034 to 0.0122

Abbreviations: β, coefficient; SE (β), standard error of β; 95% CI, confidential interval at 95%.

In the univariate analysis for CRC, mean age was the only variable significantly associated with effect size (β = −0.0256; 95% CI −0.0512 to −0.0001; *P* = .050). Publication year (β = −0.0071; 95% CI −0.0297 to 0.0156; *P* = .540) and other covariates, including number of patients, gender, median follow-up, middle year, nation, and continent, were not statistically significant (*P* > .05).

For SBC, number of patients (β = −0.00003; 95% CI −0.00005 to −8.63e-06; *P* = .007), gender (males, β = −0.00006, 95% CI −0.0001 to −0.00001, *P* = .009; and females, β = −0.00006, 95% CI −0.0001 to −0.00001, *P* = .009), mean age (β = −0.0615, 95% CI −0.0112 to −0.0111, *P* = .017), middle year (β = −0.0491, 95% CI −0.0775 to −0.00207, *P* = .001), and publication year (β = −0.0527; 95% CI: −0.0786 to −0.0268; *P* < .001) were significant predictors, except for median follow-up, nation, and continent.

In the multivariate model for CRC, mean age (β = −0.0269; 95% CI −0.0503 to −0.0035; *P* = .024) and median follow-up duration (β = −0.0321; 95% CI −0.0633 to −0.0008; *P* = .044) remained independently associated with effect size, as did middle year (β = 0.0520, 95% CI 0.0117 to 0.0923, *P* = .011) and publication (β = −0.0700, 95% CI −0.1228 to −0.0172, *P* = .009) when they were included together in the model. For SBC, mean age (β = −0.0630, 95% CI −0.1193 to −0.0067, *P* = .028) showed an association, while no association was found when middle year and publication were included together in the model.

### 3.5. Strength of epidemiological evidence

The evaluation of epidemiological evidence for cancer risk in CD patients highlighted significant variability in the strength of the estimates (Table 3). For CRC, overall risk showed high confidence in the effect estimate. The evidence for specific subtypes of CRC showed moderate strength for colonic cancer in CD and ileocolonic CD. In contrast, the evidence for rectal cancer and ileal CD was classified as low strength. When evaluating SBC, the overall risk for patients with CD was determined to be moderate, with the highest risk for SBC observed in ileocolonic and ileal CD subsets.

**Table 3.** Strength of epidemiologic evidence (ie, confidence in the estimate of effect) for inflammatory bowel disease.

Risk evidence	Colorectal cancer	Small bowel cancer
**High**	Colonic CD Ileocolonic CD Rectal cancer	
**Moderate**	CRC overall risk	SBC overall risk SBC ileocolonic SBC ileal
**Low**	Colon cancer Ileal CD	

The strength of epidemiologic evidence was rated as follows: High, if all criteria were satisfied—precision of the estimate (*P* < .001), consistency of results (*I*^2 < ^50% and Cochran Q test *P* > .10); Moderate, if a maximum of 1 criterion was not satisfied and a *P* < .001 was found; and Low, in either cases (*P* < .05).

Abbreviations: CRC, colorectal cancer; SBC, small bowel cancer; CD, Crohn’s disease.

## 4. Discussion

The present meta-analysis has revealed an increased risk of both CRC and SBC in CD. This study, the first of its kind, has examined intestinal cancer risk in CD with a substantial sample size of over 120 000 patients with CD and 500 patients with observed cancer. It exclusively included population-based cohort studies with well-defined criteria, providing a comprehensive understanding of the disease’s implications.

Only four meta-analyses have been conducted on the incidence of CRC in CD. However, three meta-analyses included a single referral center cohort, multicenter studies, and non-randomized studies. Among those publications, only the Jess^37^ meta-analysis focused solely on population-based cohort studies. This type of study is the most appropriate design for analyzing the prognosis of a given disease. Additionally, cancer diagnosis, alongside surgery and death, is one of the most clearly defined and measurable endpoints in inflammatory bowel disease (IBD).

Nevertheless, Jess’ meta-analysis included a small number of population-based studies, comprising only six studies conducted in a restricted range of countries (Canada, USA, Israel, Denmark, and Sweden). It lacked studies with large cohorts and reported a small number of observed cancer cases in patients, with an overall pooled estimated SIR of 1.9 (95% CI 1.4-2.5).^37^

Our study has gathered papers from North America, Europe, Asia, and Oceania. The populations in the studies and those conducted on the same continent and/or nation were comparable regarding the observation period. We have meticulously analyzed 27 original papers for over 125 000 patients with CD as population-based cohorts and over 500 cases of intestinal cancer, all of them matched by sex, age, and background population. The length of follow-up ranges from 2 to over 30 years, ensuring a comprehensive understanding of disease progression.

Our findings have significant implications for patient care. We observed an increased SIR compared to the general background population, with an SIR of 2.20 (95% CI 1.69-2.86). This value is more relevant than what was reported in Jess’ meta-analysis. Given the heightened risk, our results suggest a need for more personalized surveillance and care for patients with CD. In comparison to Jess’ findings,^37^ our study revealed a higher risk of CRC.

The most recent study is that by Gatenby,^30^ a New Zealand study with a follow-up of 20.4 years and an SIR of 4.1. The highest SIR is reported in Greenstein,^40^ an Israeli study with a follow-up of 11 years, showing an SIR of 6.9. Kappelman,^21^ a Danish study, had the largest cohort, 13 756 CD patients with a follow-up of 32 years and an SIR of 0.9. Only three studies, conducted in Denmark, Sweden and Italy, showed a slightly decreased risk of CRC (SIR, 0.9),^21^ CD (SIR, 0.89),^7^ and (SIR, 0.81).^28^ Interestingly, other Danish studies report low SIR of 1.1 (0.6-1.9)^5^ and 1.14 (0.31-2.92).^33^ Only one population-based cohort study, focusing on elderly-onset CD, indicated a risk of CRC with an SIR of 1.20 (95% CI 0.57-2.52).^23^ Similarly, a recent study, published during this paper’s analysis, focused solely on pediatric-onset CD and reported an SIR of 2.5 (95% CI 0.8-5.8).^39^ This disparity may be attributed to variations in geographic areas and environmental factors.

Using the methodology outlined by Jess,^37^ six studies reported separate risks of colon and rectum cancer in CD. Pooling the SIR revealed a significantly higher effect for colon cancer compared to rectal cancer.

This suggests that the higher risk of colon cancer, in comparison to rectal cancer, may be explained by the fact that colonic CD may occur without concurrent rectal inflammation. It is important to consider that patients with severe or refractory disease may have been excluded from follow-up due to surgery or death.

Information on disease extent was available, and the pooled SIR indicated an increased risk of CRC among patients with colonic CD. In patients with ileocolonic CD, the risk was further elevated, while in those with pure ileal disease, the risk was mild.

Our findings highlight that, as the number of studies, patients investigated, and follow-up periods increase, the risk of CRC is likely to be higher in patients with CD affecting the colon compared to those with pure ileal CD. The findings also suggest that colonic-only and ileocolonic CD may represent a distinct and more aggressive disease phenotype, with a greater risk of CRC development.

We observed a negative trend in the SIR of CRC for the middle year of the observation period (−2.6% per year, *P* = .05). This may be attributed to changes in disease epidemiology, chronic inflammation patterns, or environmental and genetic factors. These factors could all contribute to an elevated risk of CRC in the era of industrialization. Additionally, this trend may also reflect a growing awareness of CRC risk among patients with CD, leading to more effective diagnosis and surveillance in recent decades. It is important to note that patients with CD are often smokers,^40^^,^^41^ and that smoking increases the risk of colorectal neoplasia.^40–43^ Therefore, CD patients should be encouraged to quit smoking.

Population registries frequently lack specific data on screening frequency. The absence of detail impedes sensitivity analyses that aim to consider groups undergoing extensive surveillance. Nevertheless, the current surveillance guidelines, along with consensus and expert reviews from European and American gastroenterology societies, have largely remained consistent.^6–12^^,^^47^^,^^48^ Therefore, we assumed that patients in the included cohorts underwent monitoring with established recommendations, based on their disease onset, risk level, and medical history. Notably, only a limited number of primary studies, such as the cohort documented by Gatenby et al., explicitly delineate their surveillance methodologies. This reliance on assumptions highlights a limitation: the inherent difficulty in precisely quantifying detection bias attributable to surveillance.

As far as we know, this is the first meta-analysis that assessed the incidence of CRC plotted over time in different countries using population-based cohort studies. We performed a meta-analysis to evaluate loco-regional differences to determine whether geography influenced the natural story of the disease. Based on Figure 3, the CRC SIR in Asia and Scandinavia has remained relatively constant over the years. Notably, during the period of observation, there is an increasing trend in the CRC SIR in North America, whereas there is a declining trend in Central–Southern Europe. Additionally, an increased risk of CRC among patients with CD has been observed even in countries with well-established healthcare systems, such as North America and Scandinavia. A plausible explanation for this pattern may be related to long-term dietary changes in the Occidental–European region between the 1960s and the 2000s. During this period, Central–Southern Europe may have progressively adopted a Mediterranean dietary pattern, which is characterized by low inflammatory potential.^44–46^^,^^49^ In contrast, North America has predominantly maintained a Western-style diet that is high in saturated fats and processed foods.^50–53^ Furthermore, the use of probiotics may help reduce inflammation.^54^^,^^55^ This pro-inflammatory dietary pattern may contribute to sustained chronic inflammation and alterations in gut microbiota,^53^ thereby increasing susceptibility to CRC in individuals with CD.^51^ Although direct evidence linking these dietary transitions to CRC risk in CD patients remains limited, epidemiological and mechanistic studies consistently may support the protective role of the Mediterranean diet against inflammation and gut dysbiosis, in contrast to the well-established association between Western dietary patterns, IBD, and CRC. To our knowledge, this is the first meta-analysis that stratifies SBC according to the extent of CD in population-based cohorts. We observed an increased risk of SBC among patients with pure ileal disease and those with ileocolonic CD; in contrast, there was no clear effect on the risk for patients with colonic CD.

## 5. Conclusion

This meta-analysis highlights the strength of epidemiological evidence for the association between CD and CRC and SBC. The findings demonstrate moderate confidence in the increased overall risk of CRC and SBC, which underscores the need for vigilant cancer surveillance in patients with IBD.

Notably, specific subtypes of IBD showed varying levels of evidence for cancer risk. Colonic CD, ileocolonic CD, and rectal cancer were associated with a high strength of evidence for CRC, while ileal CD exhibited low evidence. For SBC, the ileocolonic SBC and ileal SBC presented moderate evidence. These findings emphasize the importance of differentiating disease location when assessing cancer risk in IBD populations.

The variation in the strength of evidence across different cancer types and disease subtypes probably reflects the heterogeneity in study methodologies, patient populations, and genetic or environmental factors. Future contemporary and prospective cohort studies that incorporate updated patient evaluations and modern treatment approaches are expected to offer a more accurate and current understanding of the prognosis for CD in relation to intestinal cancer risk.

The strengths of our study lie in its population-based design, which ensures thorough follow-up and effectively eliminates the risk of selection bias. Additionally, the extensive sample size of over 125 000 patients, including more than 500 documented cancer cases among those with CD, provides robust data. Furthermore, the long-term follow-up period, averaging over 10 years, allows for the derivation of specific cancer risk estimates for various cancer types in patients with CD.

Potential limitations of our study include the use of some retrospective studies and the risk of detection bias, where subclinical cancers may be detected more frequently in patients with IBD due to closer monitoring than the general population.

In conclusion, patients with pure colonic and ileocolonic CD and patients with pure ileal or ileocolonic CD need a personalized approach and stricter surveillance, respectively, for CRC and SBC.

## Supplementary Material

jjag013_Supplementary_Data

## Data Availability

The data, analytic methods, and study materials used in this research will be made available to other researchers upon request.
