# Supplementary material for: Risk of intestinal cancer in Crohn’s disease: re-analysis and meta-regression of population-based cohort studies
Source: J Crohns Colitis. 2026 Feb 28;20(2):jjag013. doi: 10.1093/ecco-jcc/jjag013 (PMC13016771; doi:10.1093/ecco-jcc/jjag013)
Supplement: jjag013_Supplementary_Data [file jjag013_supplementary_data.docx]

**Supplementary Materials**

**Poisson**

When studies reported standardized incidence ratios (SIRs), calculated as the ratio between observed and expected cases, but did not provide corresponding 95% confidence intervals (CIs), we derived the intervals based on the reported observed and expected case numbers. To ensure consistency and statistical rigor, we assumed that the observed cases followed a Poisson distribution and computed exact 95% CIs using the Garwood method. This approach is particularly suitable for rare events or small counts, as it avoids the inaccuracies of asymptotic approximations and provides conservative, exact intervals. The method has been widely adopted in epidemiological research to ensure reliable and robust interval estimation^1-4^.

**ICD tabulation**

The transition from ICD-7 to ICD-10-CM can introduce misclassification bias in SIR for CD and related cancers due to increased code granularity, which enhances case ascertainment by better capturing site-specific and complication. This leads to ascertainment bias, inflating observed cases post-transition while expected rates may lag from incomplete bridging. For malignancies, surveillance bias arises from refined topographic coding and stricter multiple-primary rules, potentially over-identifying synchronous tumors in numerators but underestimating ratios if denominators remain unadjusted **Supplementary Table 4**.

**References**

1. Ulm K. A *simple method to calculate the confidence interval of a standardized mortality ratio* (SMR). Am J Epidemiol. 1990 Feb;131(2):373-5. doi: 10.1093/oxfordjournals.aje.a115507. PMID: 2296988.
2. Sahai H, Khurshid A. *Statistics in Epidemiology: Methods, Techniques and Applications*. CRC Press; 1995.
3. Garwood F. *Fiducial limits for the Poisson distribution*. Biometrika, Volume 28, Issue 3-4, December 1936, Pages 437–442, https://doi.org/10.1093/biomet/28.3-4.437
4. Breslow NE, Day NE. *Statistical Methods in Cancer Research, Volume II – The Design and Analysis of Cohort Studies*. IARC Sci Publ. 1987;(82):1-406. PMID: 3329634.

**Supplementary Table 1**. PRISMA 2020 Checklist.

| **Section and Topic** | **Item #** | **Checklist item** | **Location where item is reported** |
| --- | --- | --- | --- |
| **TITLE** | | |  |
| Title | 1 | Identify the report as a systematic review. | 1 |
| **ABSTRACT** | | |  |
| Abstract | 2 | See the PRISMA 2020 for Abstracts checklist. | 3 |
| **INTRODUCTION** | | |  |
| Rationale | 3 | Describe the rationale for the review in the context of existing knowledge. | 4 |
| Objectives | 4 | Provide an explicit statement of the objective(s) or question(s) the review addresses. | 4 |
| **METHODS** | | |  |
| Eligibility criteria | 5 | Specify the inclusion and exclusion criteria for the review and how studies were grouped for the syntheses. | 5 |
| Information sources | 6 | Specify all databases, registers, websites, organisations, reference lists and other sources searched or consulted to identify studies. Specify the date when each source was last searched or consulted. | 5 |
| Search strategy | 7 | Present the full search strategies for all databases, registers and websites, including any filters and limits used. | 5-supplement |
| Selection process | 8 | Specify the methods used to decide whether a study met the inclusion criteria of the review, including how many reviewers screened each record and each report retrieved, whether they worked independently, and if applicable, details of automation tools used in the process. | 5-6 |
| Data collection process | 9 | Specify the methods used to collect data from reports, including how many reviewers collected data from each report, whether they worked independently, any processes for obtaining or confirming data from study investigators, and if applicable, details of automation tools used in the process. | 5-6 |
| Data items | 10a | List and define all outcomes for which data were sought. Specify whether all results that were compatible with each outcome domain in each study were sought (e.g. for all measures, time points, analyses), and if not, the methods used to decide which results to collect. | 5-6 |
|  | 10b | List and define all other variables for which data were sought (e.g. participant and intervention characteristics, funding sources). Describe any assumptions made about any missing or unclear information. | 5-6 |
| Study risk of bias assessment | 11 | Specify the methods used to assess risk of bias in the included studies, including details of the tool(s) used, how many reviewers assessed each study and whether they worked independently, and if applicable, details of automation tools used in the process. | 5-6- supplementary |
| Effect measures | 12 | Specify for each outcome the effect measure(s) (e.g. risk ratio, mean difference) used in the synthesis or presentation of results. | 5-6 |
| Synthesis methods | 13a | Describe the processes used to decide which studies were eligible for each synthesis (e.g. tabulating the study intervention characteristics and comparing against the planned groups for each synthesis (item #5)). | 5 |
|  | 13b | Describe any methods required to prepare the data for presentation or synthesis, such as handling of missing summary statistics, or data conversions. | 5-6 |
|  | 13c | Describe any methods used to tabulate or visually display results of individual studies and syntheses. | 5-6 |
|  | 13d | Describe any methods used to synthesize results and provide a rationale for the choice(s). If meta-analysis was performed, describe the model(s), method(s) to identify the presence and extent of statistical heterogeneity, and software package(s) used. | 5-6 |
|  | 13e | Describe any methods used to explore possible causes of heterogeneity among study results (e.g. subgroup analysis, meta-regression). | 5-6 |
|  | 13f | Describe any sensitivity analyses conducted to assess robustness of the synthesized results. | 5-6 |
| Reporting bias assessment | 14 | Describe any methods used to assess risk of bias due to missing results in a synthesis (arising from reporting biases). | Supplementary 1 |
| Certainty assessment | 15 | Describe any methods used to assess certainty (or confidence) in the body of evidence for an outcome. | 5 |
| **RESULTS** | | |  |
| Study selection | 16a | Describe the results of the search and selection process, from the number of records identified in the search to the number of studies included in the review, ideally using a flow diagram. | 7-9 |
|  | 16b | Cite studies that might appear to meet the inclusion criteria, but which were excluded, and explain why they were excluded. | 7-9 |
| Study characteristics | 17 | Cite each included study and present its characteristics. | 7-9 |
| Risk of bias in studies | 18 | Present assessments of risk of bias for each included study. | Supplementary 1 |
| Results of individual studies | 19 | For all outcomes, present, for each study: (a) summary statistics for each group (where appropriate) and (b) an effect estimate and its precision (e.g. confidence/credible interval), ideally using structured tables or plots. | 7-9 |
| Results of syntheses | 20a | For each synthesis, briefly summarise the characteristics and risk of bias among contributing studies. | 7-9 |
|  | 20b | Present results of all statistical syntheses conducted. If meta-analysis was done, present for each the summary estimate and its precision (e.g. confidence/credible interval) and measures of statistical heterogeneity. If comparing groups, describe the direction of the effect. | 7-9 |
|  | 20c | Present results of all investigations of possible causes of heterogeneity among study results. | 7-9 |
|  | 20d | Present results of all sensitivity analyses conducted to assess the robustness of the synthesized results. | 7-9 |
| Reporting biases | 21 | Present assessments of risk of bias due to missing results (arising from reporting biases) for each synthesis assessed. | Supplementary 1 |
| Certainty of evidence | 22 | Present assessments of certainty (or confidence) in the body of evidence for each outcome assessed. | 7-9 |
| **DISCUSSION** | | |  |
| Discussion | 23a | Provide a general interpretation of the results in the context of other evidence. | 9-12 |
|  | 23b | Discuss any limitations of the evidence included in the review. | 12 |
|  | 23c | Discuss any limitations of the review processes used. | 12 |
|  | 23d | Discuss implications of the results for practice, policy, and future research. | 12 |
| **OTHER INFORMATION** | | |  |
| Registration and protocol | 24a | Provide registration information for the review, including register name and registration number, or state that the review was not registered. | 3 |
|  | 24b | Indicate where the review protocol can be accessed, or state that a protocol was not prepared. | 3 |
|  | 24c | Describe and explain any amendments to information provided at registration or in the protocol. | 3 |
| Support | 25 | Describe sources of financial or non-financial support for the review, and the role of the funders or sponsors in the review. | 1 |
| Competing interests | 26 | Declare any competing interests of review authors. | 1 |
| Availability of data, code and other materials | 27 | Report which of the following are publicly available and where they can be found: template data collection forms; data extracted from included studies; data used for all analyses; analytic code; any other materials used in the review. | 1-2 |

*From:*  Page MJ, McKenzie JE, Bossuyt PM, Boutron I, Hoffmann TC, Mulrow CD, et al. The PRISMA 2020 statement: an updated guideline for reporting systematic reviews. BMJ 2021;372:n71. doi: 10.1136/bmj.n71

**Supplementary Table 2.** MOOSE (Meta-analyses Of Observational Studies in Epidemiology) Checklist.

| **Reporting Criteria** | **Reported (Yes/No)** | | | | | **Reported on Page No.** | | | | |
| --- | --- | --- | --- | --- | --- | --- | --- | --- | --- | --- |
| **Reporting of Background** |  | | | | |  | | | | |
| Problem definition |  | Yes | |  | |  | | 4 | |  |
| Hypothesis statement |  | Yes | |  | |  | | 4 | |  |
| Description of Study Outcome(s) |  | Yes | |  | |  | | 4 | |  |
| Type of exposure or intervention used |  | Yes | |  | |  | | 5 | |  |
| Type of study design used |  | Yes | |  | |  | | 5 | |  |
| Study population |  | Yes | |  | |  | | 5 | |  |
| **Reporting of Search Strategy** |  | | | | |  | | | | |
| Qualifications of searchers (eg, librarians  and investigators) | Yes | | | | | 5- supplementary | | | | |
| Search strategy, including time period  included in the synthesis and keywords | Yes | | | | | 5- supplementary | | | | |
| Effort to include all available studies,  including contact with authors | Yes | | | | |  | | | | |
|  |  |  |  |  |  |  | | 5 | |  |
| Databases and registries searched |  | Yes | |  | |  | | 5-7 | |  |
| Search software used, name and  version, including special features used (e.g., explosion) | Yes | | | | | 5 | | | | |
| Use of hand searching (e.g., reference  lists of obtained articles) | Yes | | | | | 5 | | | | |
| List of citations located and those  excluded, including justification | Yes | | | | | 5 | | | | |
| Method for addressing articles  published in languages other than English | Yes | | | | | 5 | | | | |
| Method of handling abstracts and  unpublished studies | Yes | | | | | 5 | | | | |
| Description of any contact with authors |  | Yes | |  | |  | | 5 | |  |
| **Reporting of Methods** |  | | | | |  | | | | |
| Description of relevance or appropriateness of studies assembled for  assessing the hypothesis to be tested | Yes | | | | | 5 | | | | |
| Rationale for the selection and coding of data (e.g., sound clinical principles or  convenience) | Yes | | | | | 5 | | | | |
| Documentation of how data were classified and coded (e.g., multiple raters,  blinding, and interrater reliability) | Yes | | | | | supplementary | | | | |
| Assessment of confounding (eg, comparability of cases and controls in  studies where appropriate | Yes | | | | | supplementary | | | | |
| **Reporting Criteria** | **Reported (Yes/No)** | | | | | **Reported on Page No.** | | | | |
| Assessment of study quality, including blinding of quality assessors; stratification or regression on possible  predictors of study results | Yes | | | | | supplementary | | | | |
| Assessment of heterogeneity |  | | Yes | |  |  | 6 | |  | |
| Description of statistical methods (e.g.,  complete description of fixed or random effects models, justification of whether the chosen models account for predictors of study results, dose-response models, or cumulative meta-analysis) in sufficient  detail to be replicated | Yes | | | | | 6 | | | | |
| Provision of appropriate tables and  graphics | Yes | | | | | 14-21 | | | | |
| **Reporting of Results** |  | | | | |  | | | | |
| Table giving descriptive information for  each study included | Yes | | | | | 19 | | | | |
| Results of sensitivity testing (e.g.,  subgroup analysis) | Yes | | | | | 6-8 | | | | |
| Indication of statistical uncertainty of  Findings | Yes | | | | | 7 | | | | |
| **Reporting of Discussion** |  | | | | |  | | | | |
| Quantitative assessment of bias (e.g.,  publication bias) | Yes | | | | | supplementary | | | | |
| Justification for exclusion (e.g., exclusion  of non–English-language citations) | No | | | | | N/A | | | | |
| Assessment of quality of included studies |  | | Yes | |  |  | supplementary | |  | |
| **Reporting of Conclusions** |  | | | | |  | | | | |
| Consideration of alternative explanations  for observed results | Yes | | | | | 9-10 | | | | |
| Generalization of the conclusions (i.e., appropriate for the data presented and  within the domain of the literature review) | Yes | | | | | 11-12 | | | | |
| Guidelines for future research |  | | Yes | |  |  | 12 | |  | |
| Disclosure of funding source |  | | Yes | |  |  | 2 | |  | |

**Search Strategy**

PUBMED

((Intestinal Neoplasms[MeSH Terms]) OR (Intestin* Neoplasm*)) OR (Intestin* cancer) OR (Cecal Neoplasms[MeSH Terms]) OR (Cecal Neoplasm*) OR (Cecal cancer) OR (cecum cancer) OR (caec* neoplasm*) OR (caec* cancer) OR (Appendiceal Neoplasms[MeSH Terms]) OR (Appendi* Neoplasm*) OR (appendi* cancer) OR (Colorectal Neoplasms[MeSH Terms]) OR (Colorectal Neoplasm*) OR (Colorectal cancer) OR (Colonic Neoplasms[MeSH Terms]) OR (Colonic Neoplasm*) OR ("colon cancer") OR (Colon Adenocarcinoma) OR (Colon Neoplasm*) OR (Sigmoid Neoplasms[MeSH Terms]) OR (Sigmoid Neoplasm*) OR (Sigmoid cancer) OR ("Sigmoid Colon Cancer") OR (Adenomatous Polyposis Coli[MeSH Terms]) OR (Adenomatous Polyposis Coli) OR ("Familial Adenomatous Polyposis Coli") OR (Colorectal Neoplasms, Hereditary Nonpolyposis[MeSH Terms]) OR (Colorectal Neoplasms, Hereditary Nonpolyposis) OR (Familial Nonpolyposis Colon Cancer) OR (Hereditary Nonpolyposis Colorectal Neoplasms) OR (Lynch Syndrome I) OR (Hereditary Nonpolyposis Colorectal Cancer) OR (Rectal Neoplasms[MeSH Terms]) OR (Rect* Neoplasm*) OR (rect* cancer) OR (rect* tumor) OR (Ileal Neoplasms[MeSH Terms]) OR (Ileal Neoplasm*) OR (Ileal cancer) OR (Ileum neoplasm*) OR (ileum cancer)AND("crohn disease"[MeSH Terms] OR ("crohn"[All Fields] AND "disease"[All Fields]) OR "crohn disease"[All Fields] OR "crohn s disease"[All Fields] OR ("crohn disease"[MeSH Terms] OR ("crohn"[All Fields] AND "disease"[All Fields]) OR "crohn disease"[All Fields])) AND ("population*"[All Fields] OR "individual*"[All Fields] OR "Persons"[MeSH Terms]) AND ("Risk"[MeSH Terms] OR "Risk"[All Fields] OR "factor*"[All Fields] OR "probabilit*"[All Fields] OR "predict*"[All Fields] OR "Risk"[MeSH Terms])

Filter: only human

EMBASE

((**'intestine tumor'** OR **'intestine tumor'**:ti,ab,kw OR **'intestine tumor'**/exp) AND **'crohn disease'** OR **'crohn disease'**:ti,ab,kw OR **'crohn disease'**/exp) AND **'risk factor'** AND  (**'case control study'**/de OR **'cohort analysis'**/de OR **'controlled clinical trial'**/de OR **'controlled study'**/de OR **'multicenter study'**/de OR **'observational study'**/de OR **'population based case control study'**/de OR **'prospective study'**/de OR **'randomized controlled trial'**/de OR **'retrospective study'**/de OR ‘cross sectional study’/de) AND ‘human’/de

SCOPUS

( ALL ( intestinal AND cancer ) OR TITLE-ABS-KEY ( intestinal AND cancer ) AND ALL ( crohn's AND disease ) OR TITLE-ABS-KEY ( crohn's AND disease ) AND ALL ( risk AND factor ) )

Filter: SUBJECT Limited to Medicine;

**Supplementary Table 3.** Summary of quality score using the modified Newcastle-Ottawa Scale.


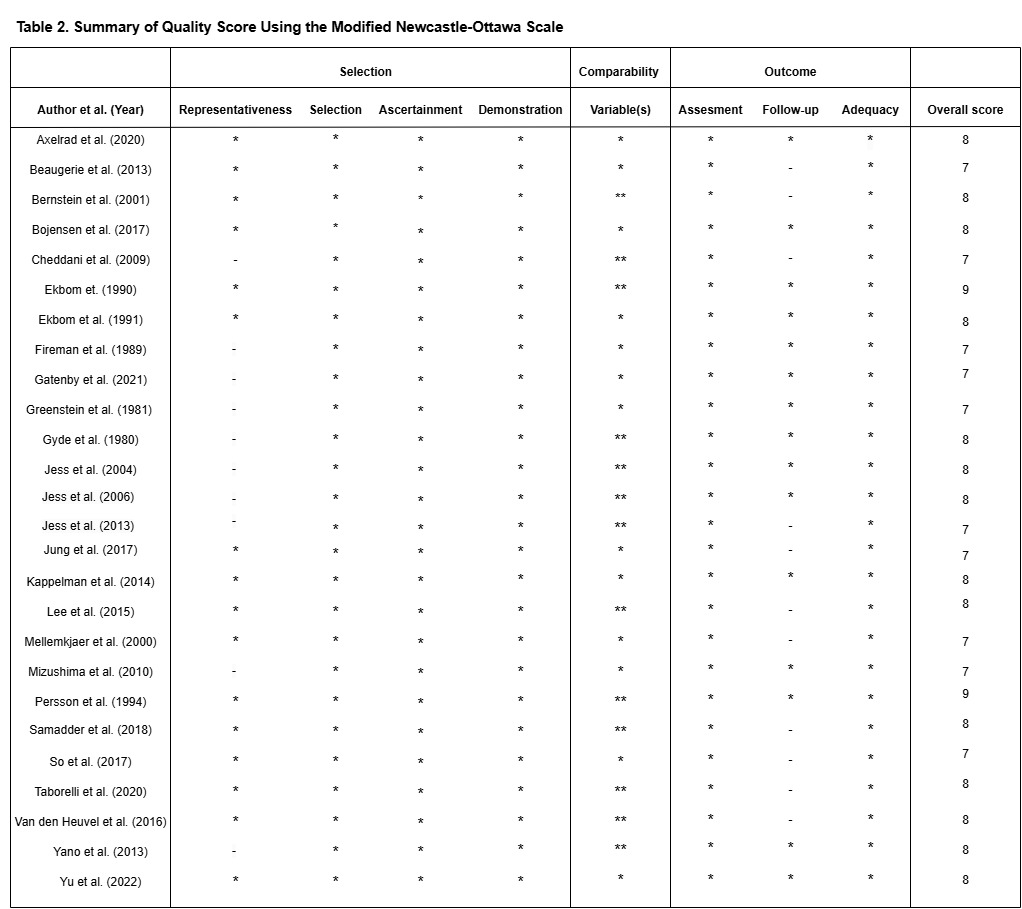


| **Disease** | **ICD-7** | **ICD-8** | **ICD‑9-CM** | **ICD‑10-CM** | **Note** |
| --- | --- | --- | --- | --- | --- |
| Crohn’s disease (General) | 572.0 | 563.00 | 555.9 | K50.90 | Crohn's disease, unspecified, without complications |
| Crohn’s disease of small intestine | 572.0 | 563.00 | 555.0 | K50.00 | Duodenum, Ileum or jejunum |
| Crohn’s disease of large intestine | 572.1 | 563.01 | 555.1 | K50.10 | Bowel, colon, or rectum |
| *Malignant neoplasm* |  |  |  |  |  |
| Malignant neoplasm of small intestine | 152 | 152 | 152.9-152.9 | C17.0-C17.9 | Unspecified site |
| Colon cancer (malignant) | 153 | 153 | 153.0-153.9 | C18.0-C18.9 | Unspecified site |
| Rectal / rectosigmoid cancer | 154 | 154 | 154.0-154.1 | C19.0-C20.0 | -- |
| Anal canal/Anus cancer | 154 | 154 | 154.2-154.3 | C21.0-C21.9 | -- |
| Hereditary non-polyposis colon cancer (family history) | - | - | V16.0 | Z80.0 | Family history of malignant neoplasm of digestive organs, not actual |

**Supplementary Table 4.** Crohn’s disease and Cancer ICD tabulation


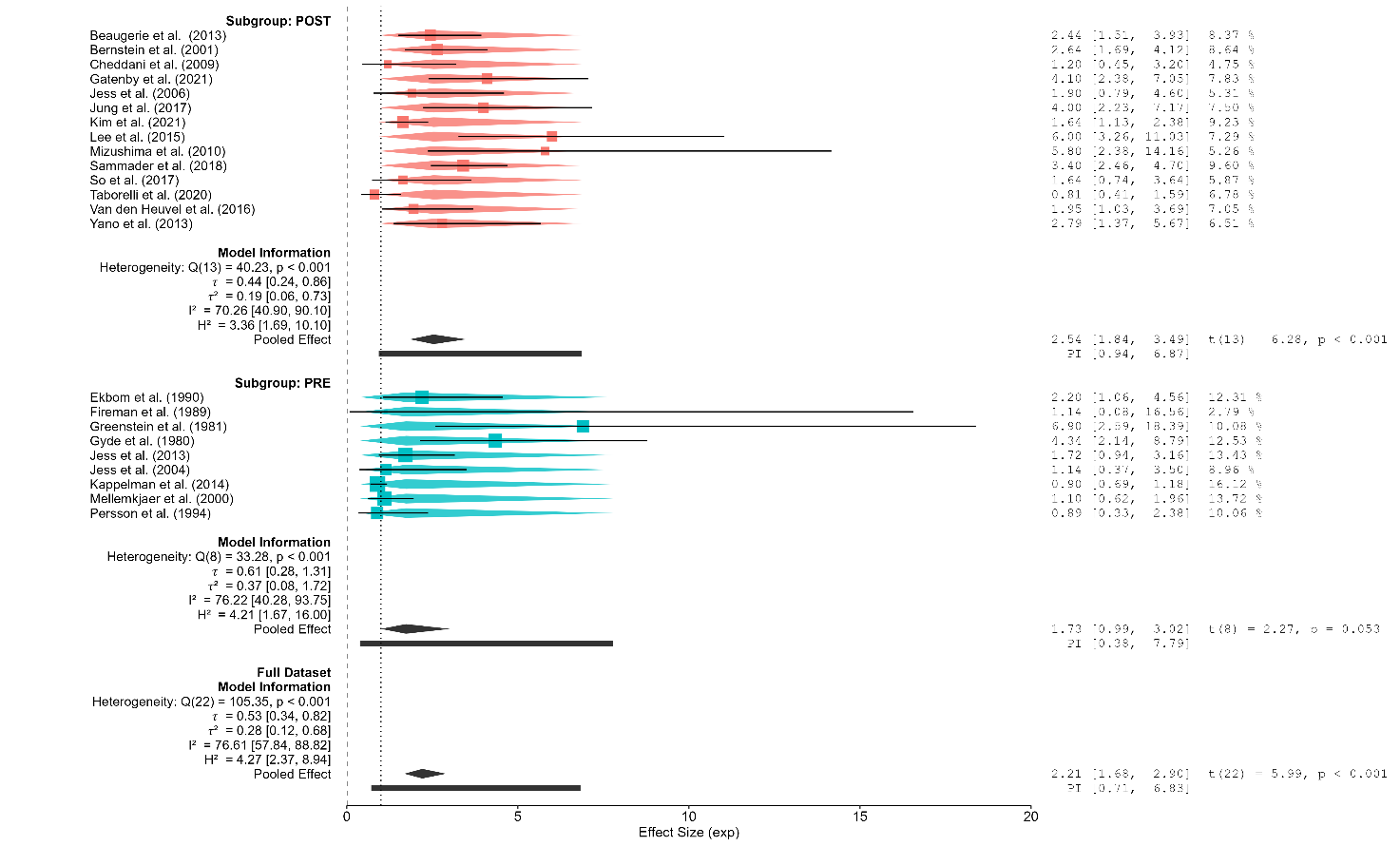


**Supplementary Figure 1.** SIR with 95% C.I. for colorectal cancer in Crohn’s Disease (log scale) in separated estimation pre and post 1997. Individual and combined standardized incidence ratios (with 95% confidence intervals) of colorectal cancer in Crohn’s Disease. The size of the boxes is proportional to the weight (1/SE) of each study.


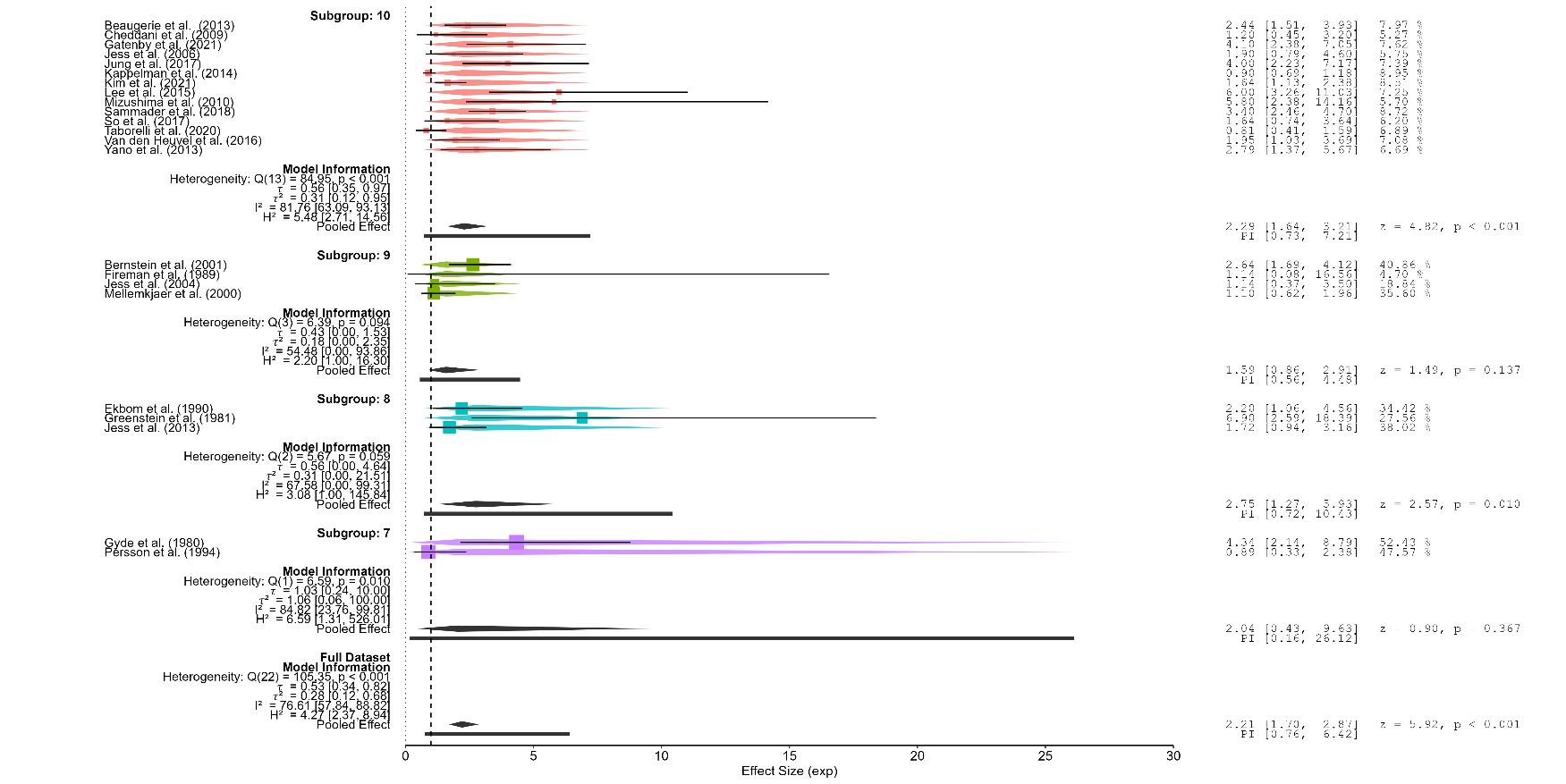


**Supplementary Figure 2.** SIR with 95% C.I. for colorectal cancer in Crohn’s Disease (log scale) in separated estimation for ICD middle year. Individual and combined standardized incidence ratios (with 95% confidence intervals) of colorectal cancer in Crohn’s Disease. The size of the boxes is proportional to the weight (1/SE) of each study.


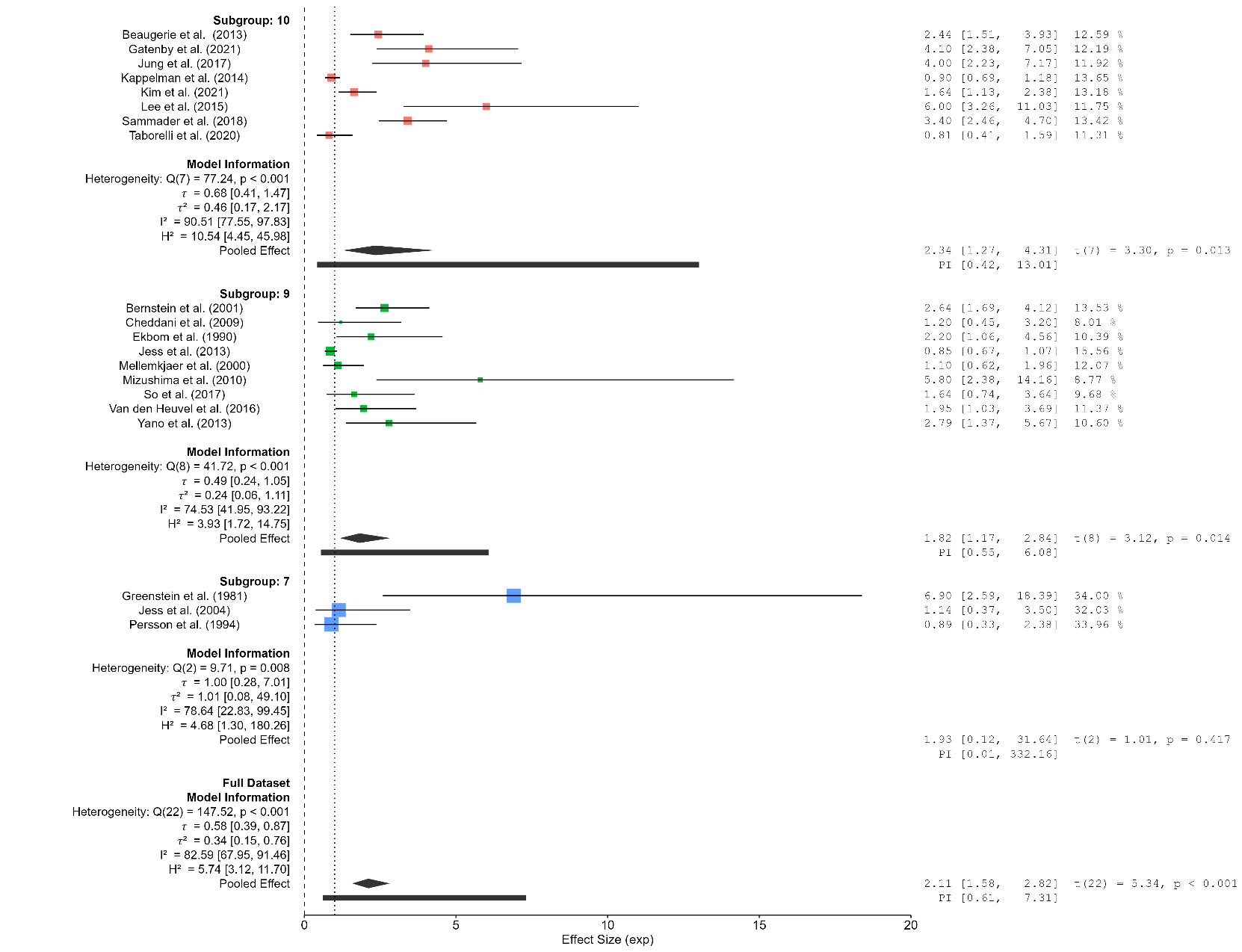


**Supplementary Figure 3.** SIR with 95% C.I. for colorectal cancer in Crohn’s Disease (log scale) in separated estimation by ICD at the beginning of the study. Individual and combined standardized incidence ratios (with 95% confidence intervals) of colorectal cancer in Crohn’s Disease. The size of the boxes is proportional to the weight (1/SE) of each study.


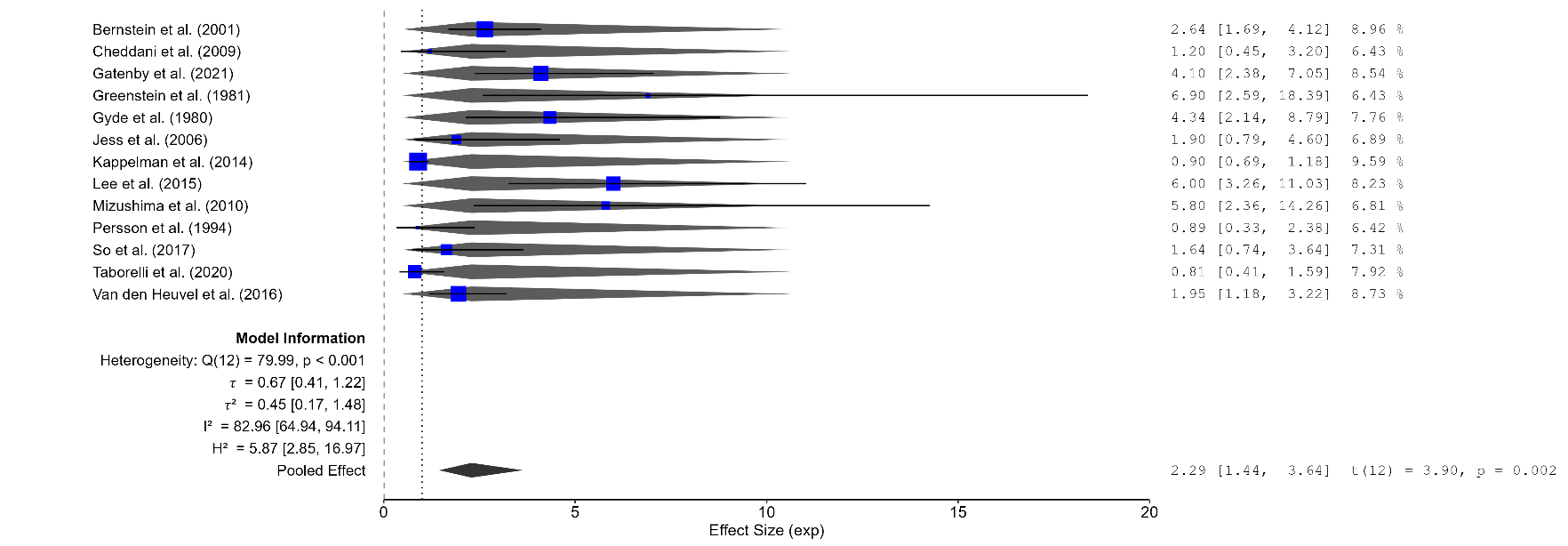


**Supplementary Figure 4.** SIR with 95% C.I. for colorectal cancer in Crohn’s Disease (log scale) in separated estimation for each nation by follow-up. Individual and combined standardized incidence ratios (with 95% confidence intervals) of colorectal cancer in Crohn’s Disease. The size of the boxes is proportional to the weight (1/SE) of each study.


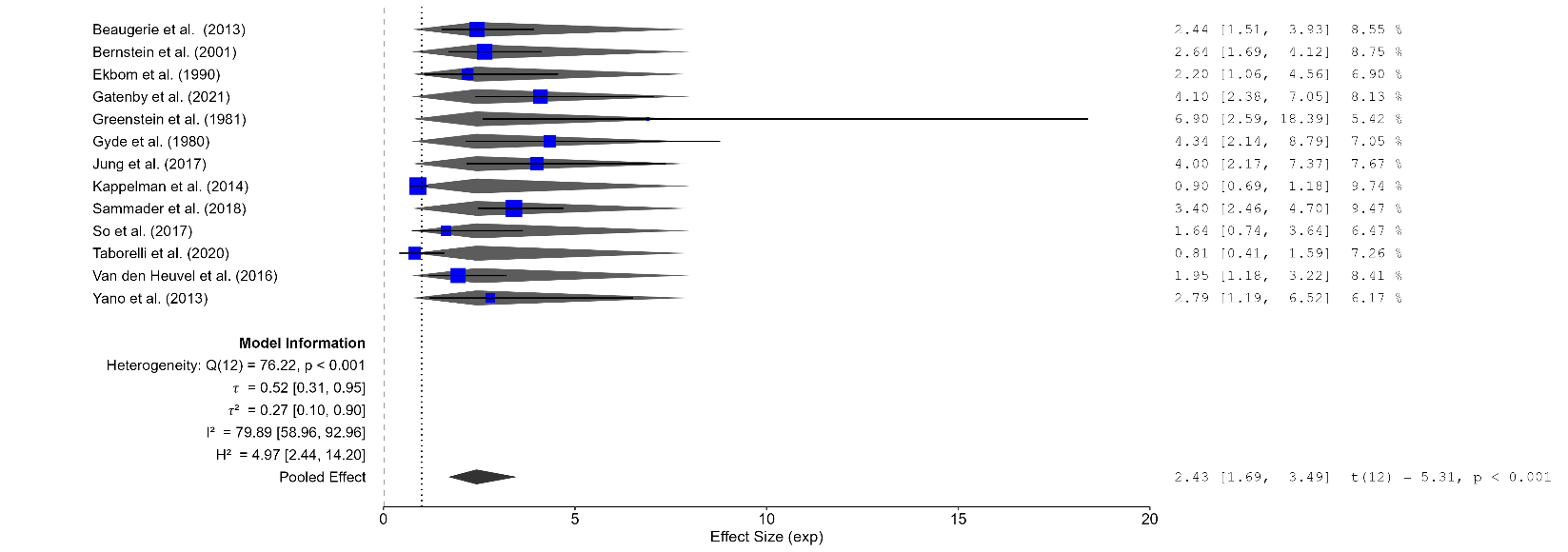


**Supplementary Figure 5.** SIR with 95% C.I. for colorectal cancer in Crohn’s Disease (log scale) in separated estimation for each nation by major observed cases. Individual and combined standardized incidence ratios (with 95% confidence intervals) of colorectal cancer in Crohn’s Disease. The size of the boxes is proportional to the weight (1/SE) of each study.


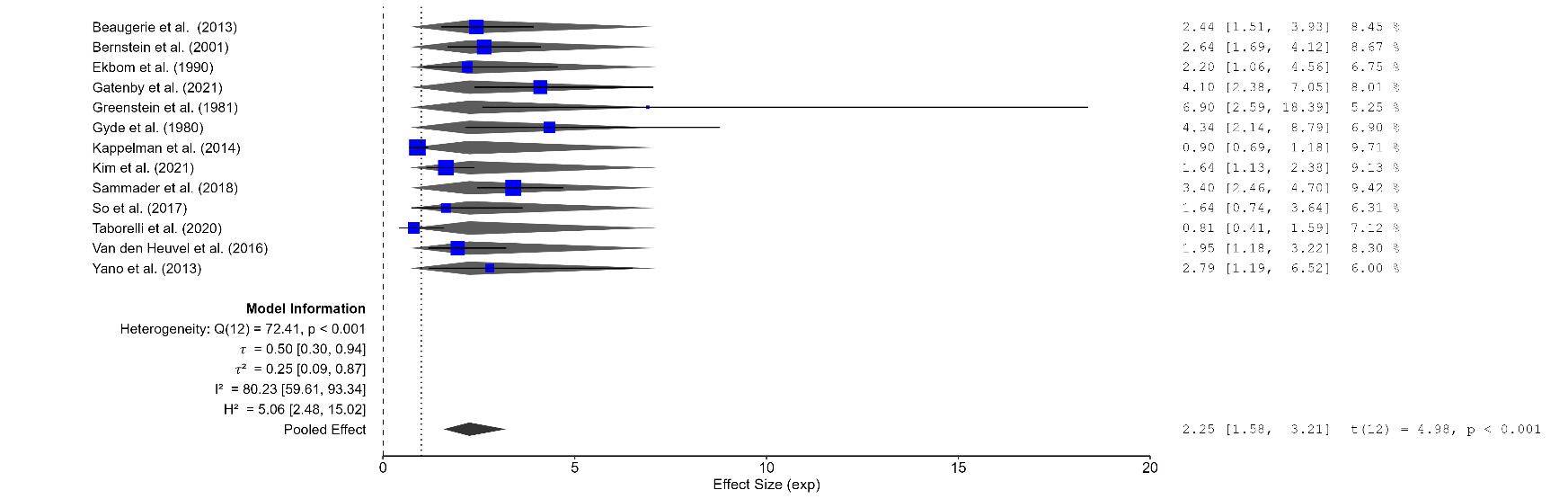


**Supplementary Figure 6.** SIR with 95% C.I. for colorectal cancer in Crohn’s Disease (log scale) in separated estimation for each nation by major population. Individual and combined standardized incidence ratios (with 95% confidence intervals) of colorectal cancer in Crohn’s Disease. The size of the boxes is proportional to the weight (1/SE) of each study.


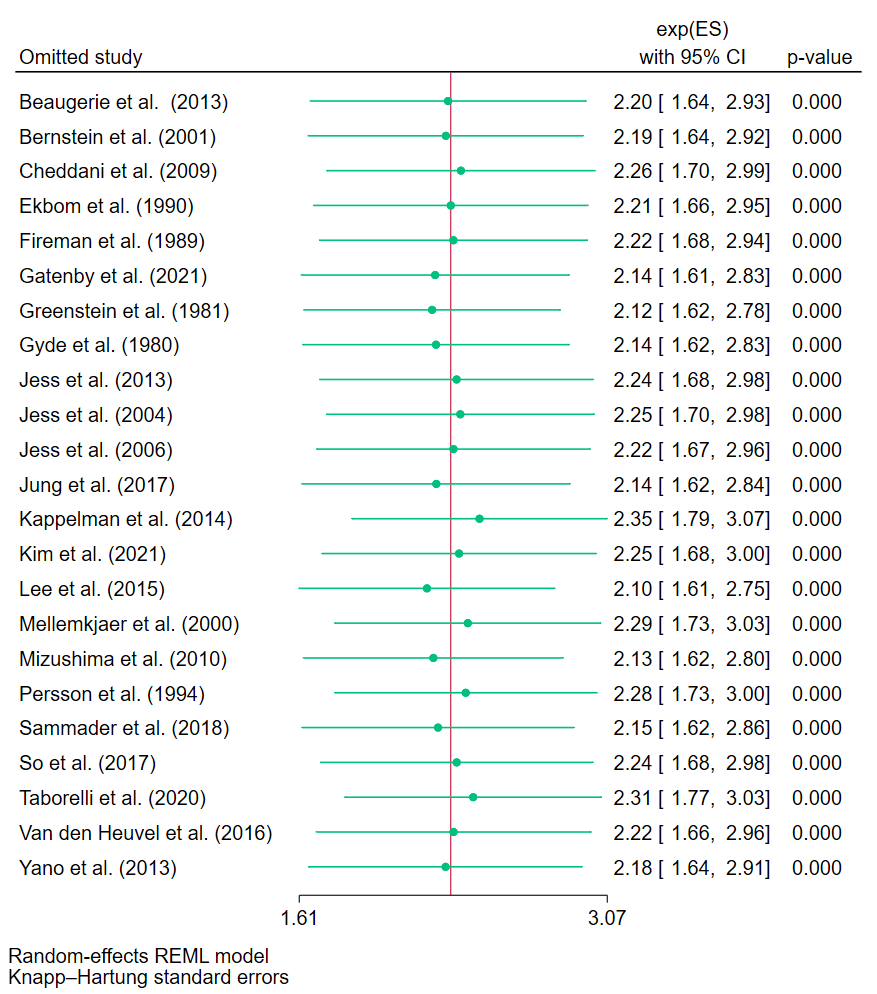


**Supplementary Figure 7.** Leave-one-out sensitivity analysis of the meta-analysis in CRC. For each included study, the pooled effect estimate (exp(ES)) and its Confidence Interval at 95% are reported. Effect estimates were obtained using a random-effects model fitted by restricted maximum likelihood (REML) with Knapp–Hartung adjustment for standard errors.


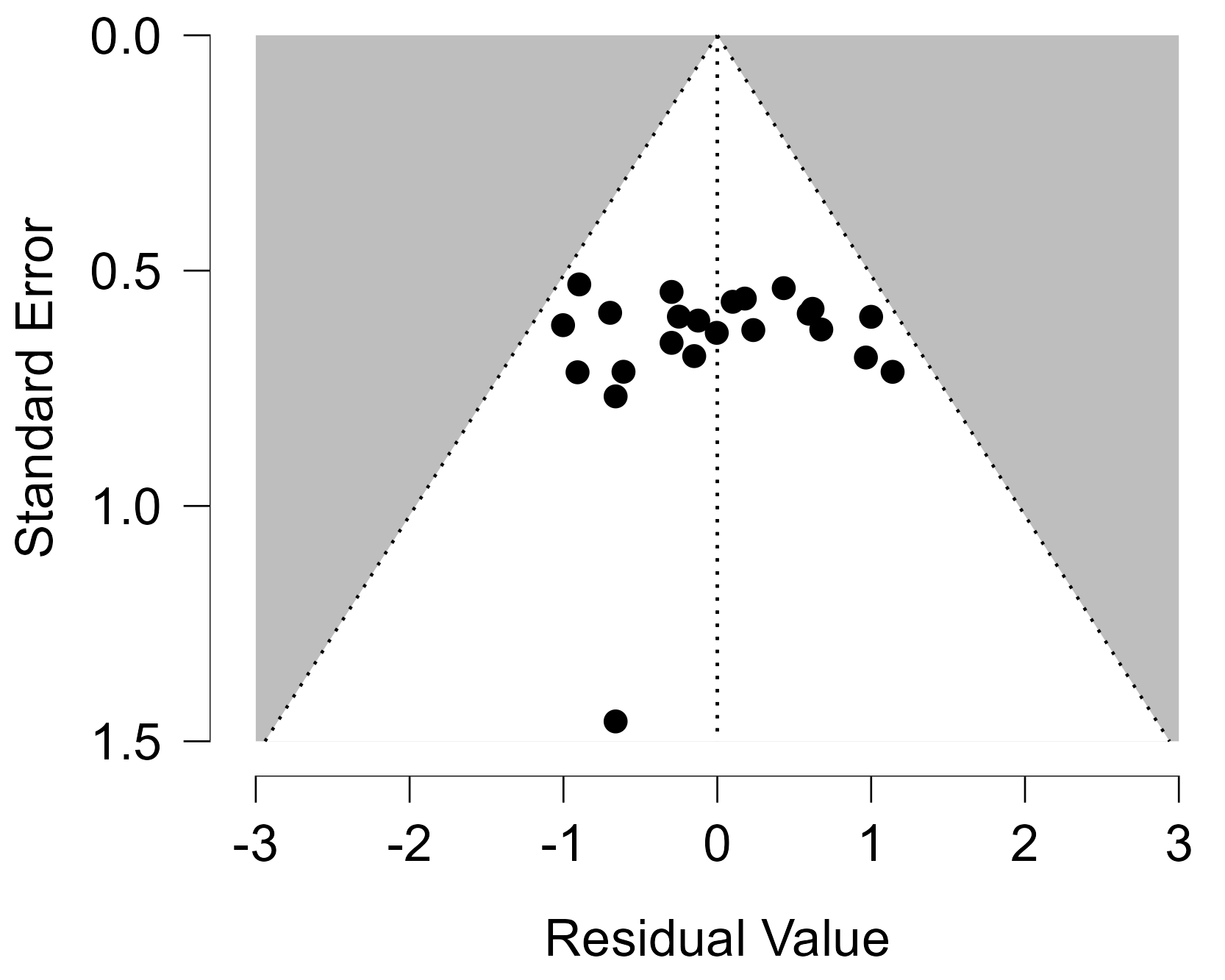


**Supplementary Figure 8.** Funnel plot CRC. Each dot represents a single study, plotted with its effect size on the x-axis and its standard error on the y-axis. The vertical red line represents the overall effect estimate derived from the random-effects model using restricted maximum likelihood (REML). The pseudo 95% confidence interval region, shown as gray diagonal lines, outlines the expected spread of studies in the absence of bias or between-study heterogeneity.


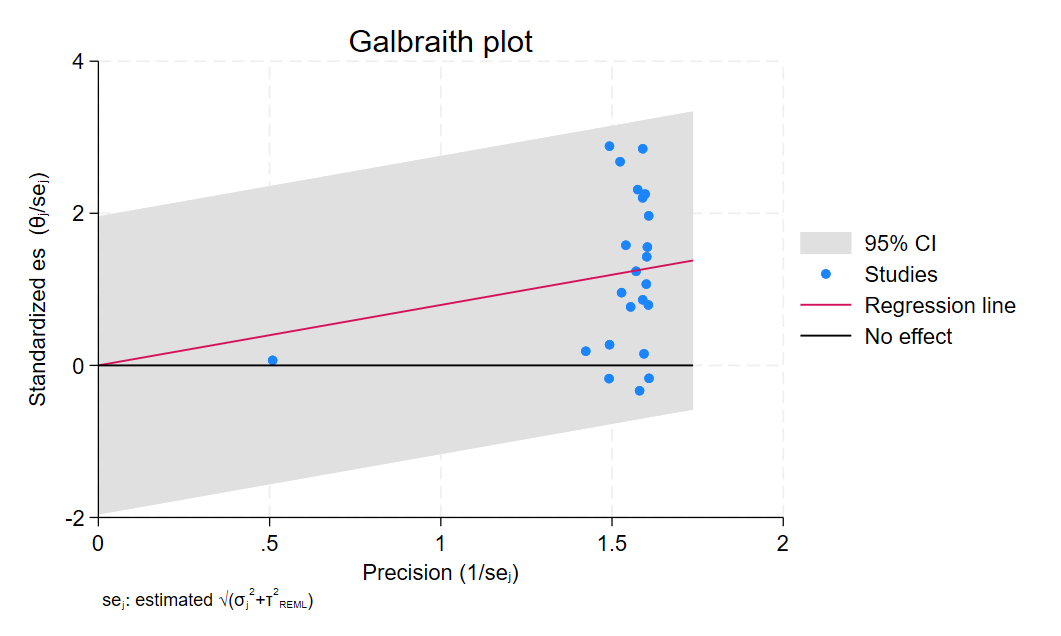


**Supplementary Figure 9.** Galbraith plot CRC. Each study is represented as a point, where the x-axis denotes the precision (defined as the inverse of the standard error), and the y-axis indicates the standardized effect size. The red regression line represents the overall summary effect estimated from the random-effects model, and the gray shaded area corresponds to the confidence interval at 95% around regression line. The horizontal black line at y = 0 corresponds to the line of no effect, serving as a reference.


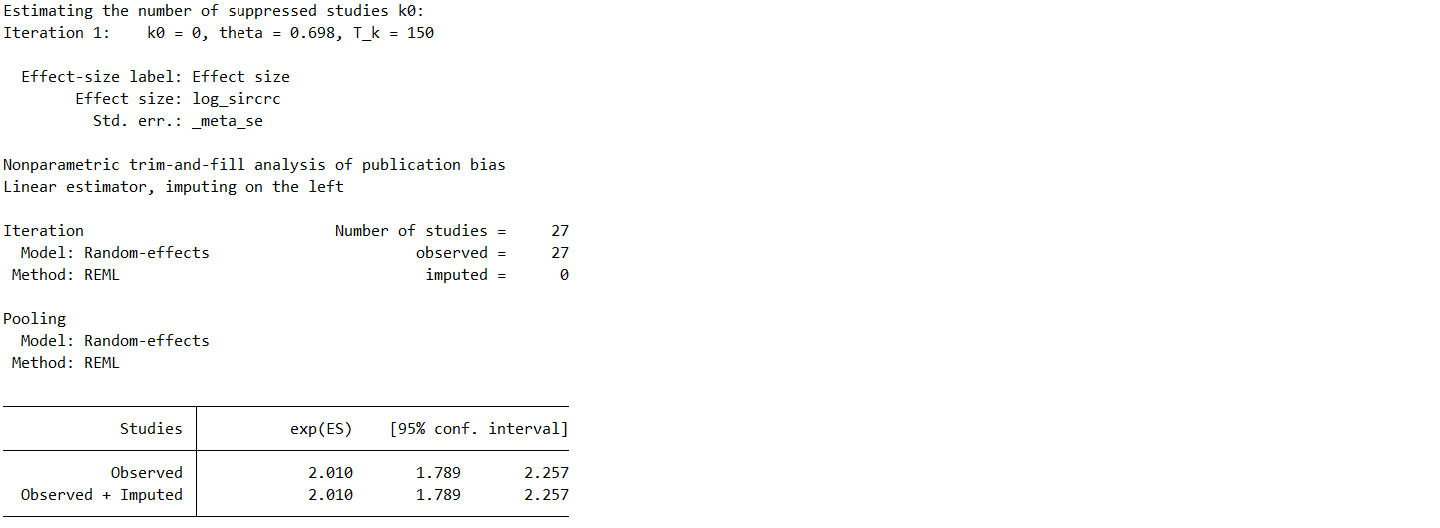


**Supplementary Figure 10.** Trim-and-fill analysis for assessment of publication bias CRC. A nonparametric trim-and-fill procedure was applied using a random-effects model estimated by restricted maximum likelihood (REML) to evaluate the potential impact of publication bias.


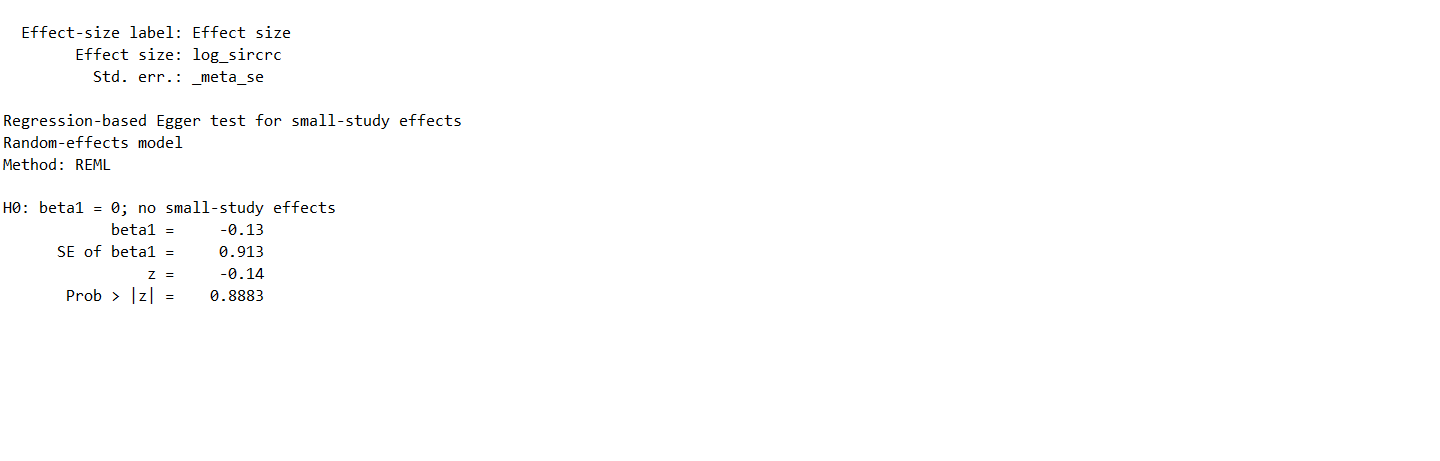


**Supplementary Figure 11.** Egger’s test for small-study effects in a meta-analysis in CRC. A random-effects model was fitted using the REML method.


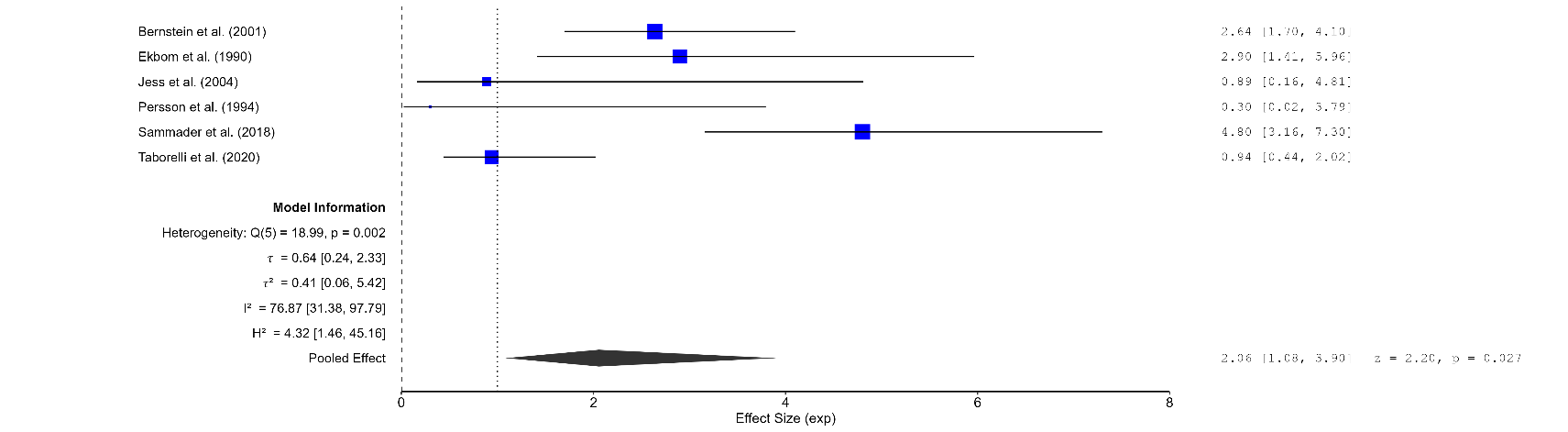


**Supplementary Figure 12.** SIR with 95% C.I. for colon cancer in Crohn’s Disease (log scale). Individual and combined standardized incidence ratios (with 95% confidence intervals) of colorectal cancer in Crohn’s Disease. The size of the boxes is proportional to the weight (1/SE) of each study.


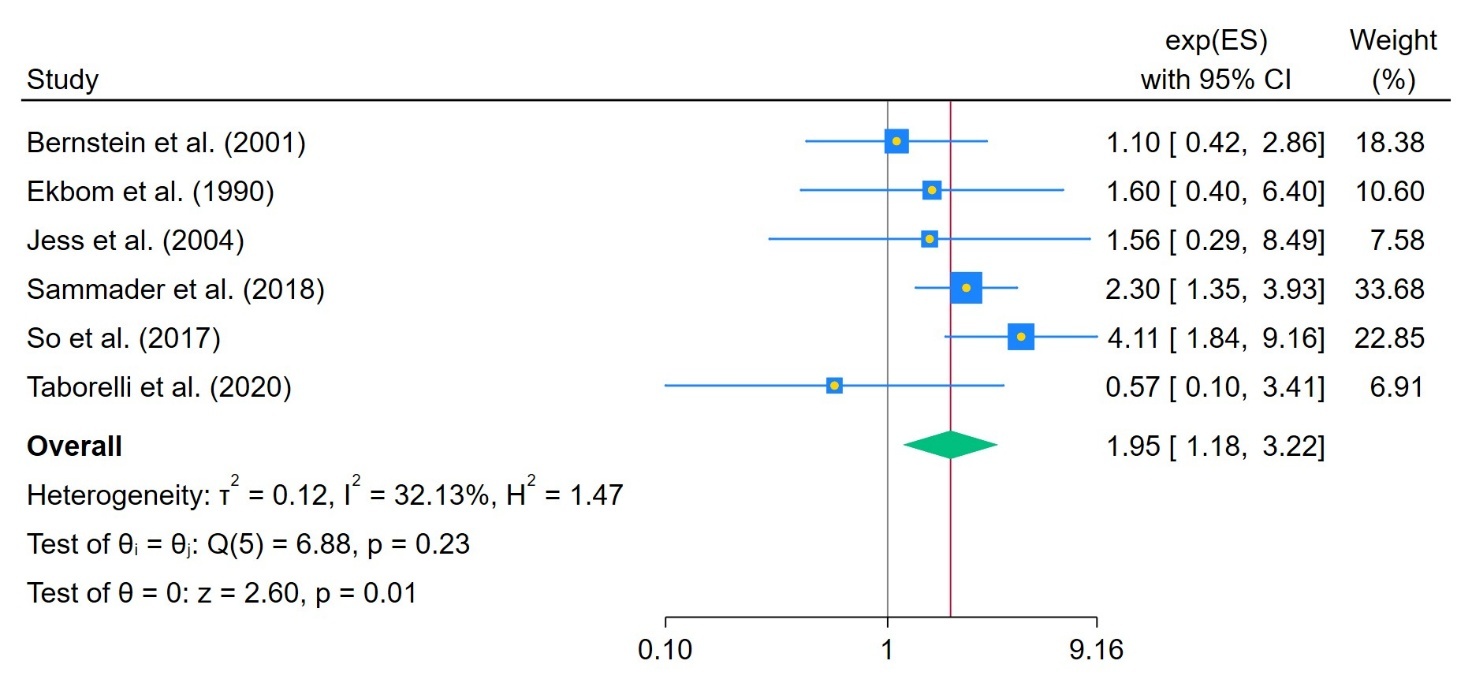


**Supplementary Figure 13.** SIR with 95% C.I. for rectal cancer in Crohn’s Disease (log scale). Individual and combined standardized incidence ratios (with 95% confidence intervals) of colorectal cancer in Crohn’s Disease. The size of the boxes is proportional to the weight (1/SE) of each study.


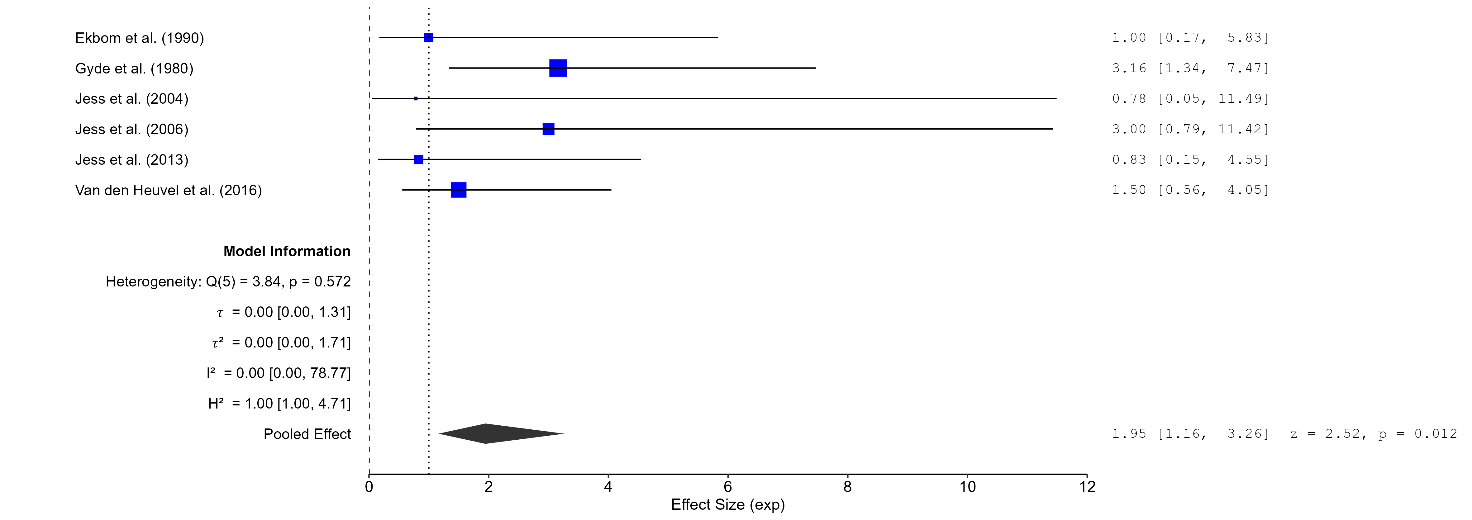


**Supplementary Figure 14.** SIR with 95% C.I. for L1 cancer in Crohn’s Disease (log scale) in separated estimation. Individual and combined standardized incidence ratios (with 95% confidence intervals) of colorectal cancer in Crohn’s Disease. The size of the boxes is proportional to the weight (1/SE) of each study.


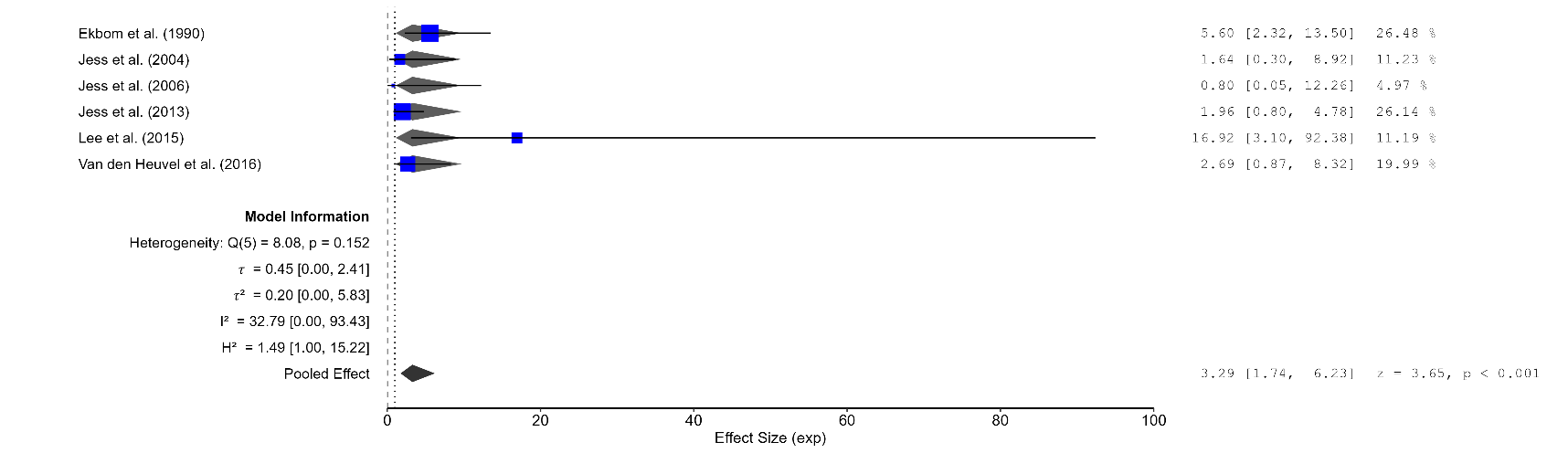


**Supplementary Figure 15.** SIR with 95% C.I. for L2 cancer in Crohn’s Disease (log scale) in separated estimation. Individual and combined standardized incidence ratios (with 95% confidence intervals) of colorectal cancer in Crohn’s Disease. The size of the boxes is proportional to the weight (1/SE) of each study.


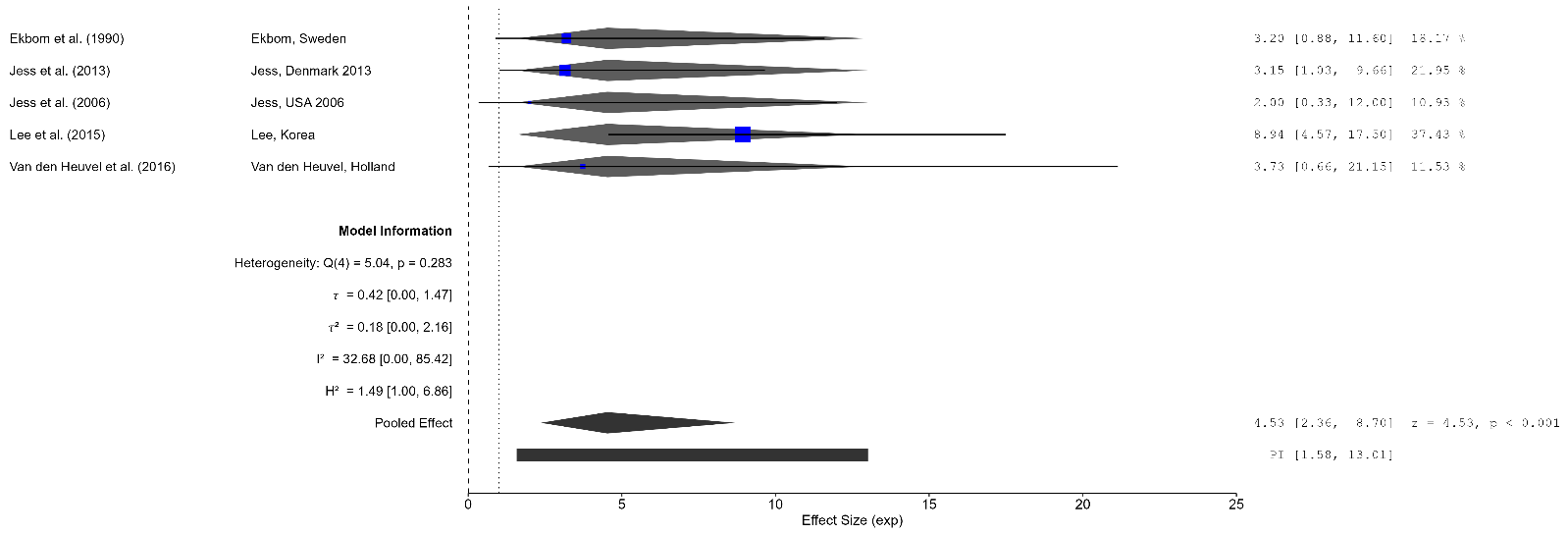


**Supplementary Figure 16.** SIR with 95% C.I. for L3 cancer in Crohn’s Disease (log scale) in separated estimation. Individual and combined standardized incidence ratios (with 95% confidence intervals) of colorectal cancer in Crohn’s Disease. The size of the boxes is proportional to the weight (1/SE) of each study.


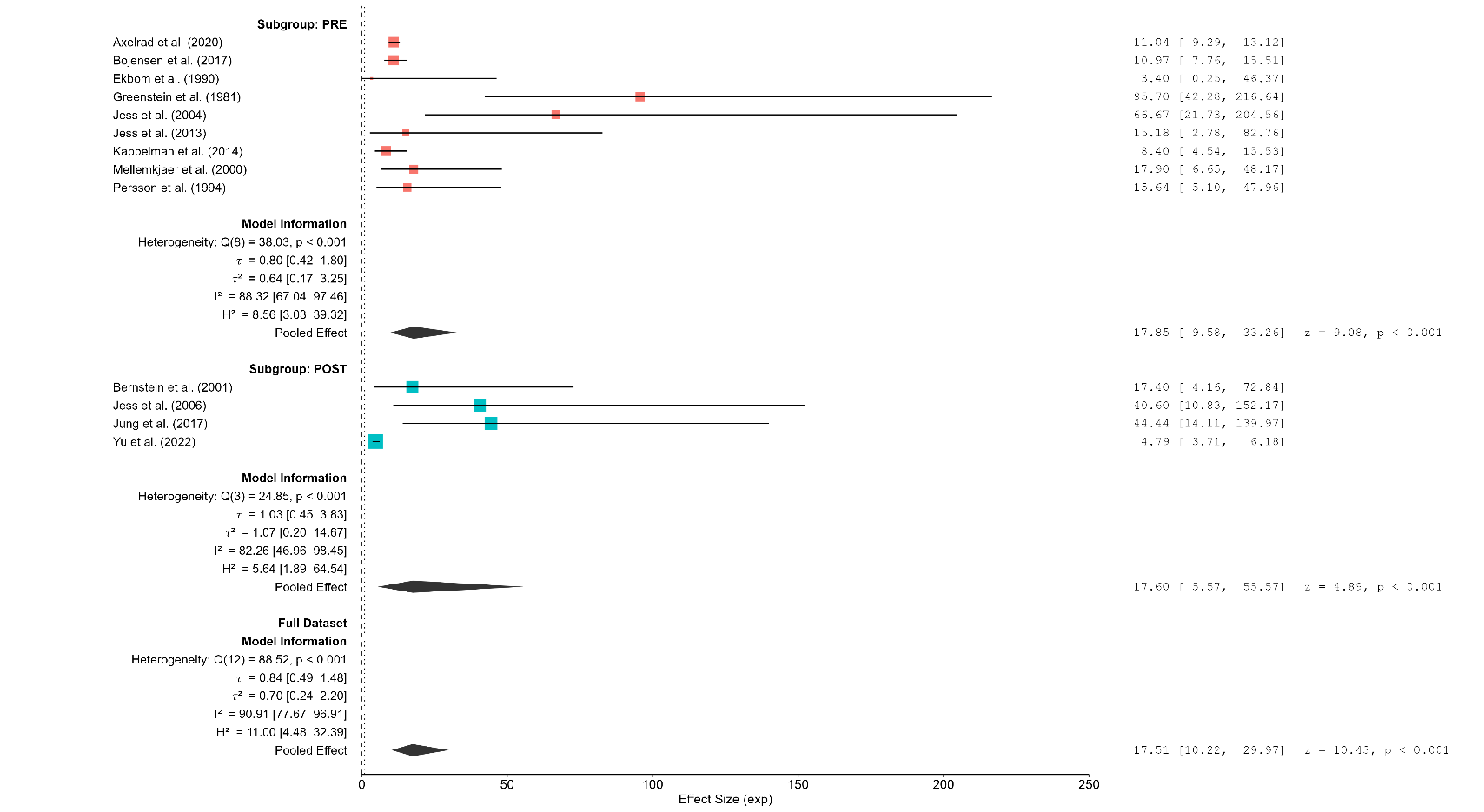


**Supplementary Figure 17.** SIR SBC with 95% C.I. cancer in Crohn’s Disease (log scale) in separated estimation pre and post 1997. Individual and combined standardized incidence ratios (with 95% confidence intervals) of colorectal cancer in Crohn’s Disease. The size of the boxes is proportional to the weight (1/SE) of each study.


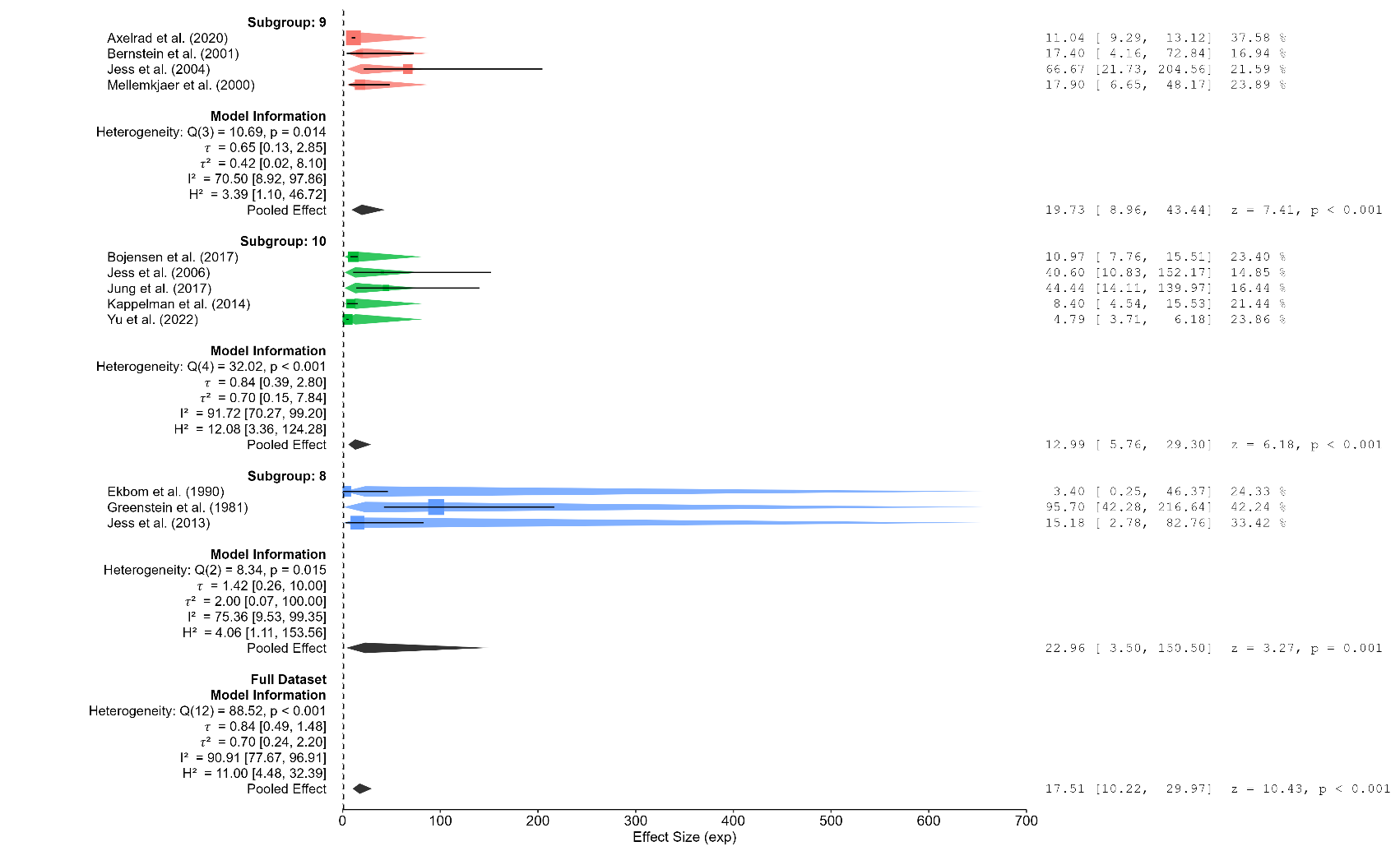


**Supplementary Figure 18.** SIR SBC with 95% C.I. cancer in Crohn’s Disease (log scale) in separated estimation for ICD middle year. Individual and combined standardized incidence ratios (with 95% confidence intervals) of colorectal cancer in Crohn’s Disease. The size of the boxes is proportional to the weight (1/SE) of each study.


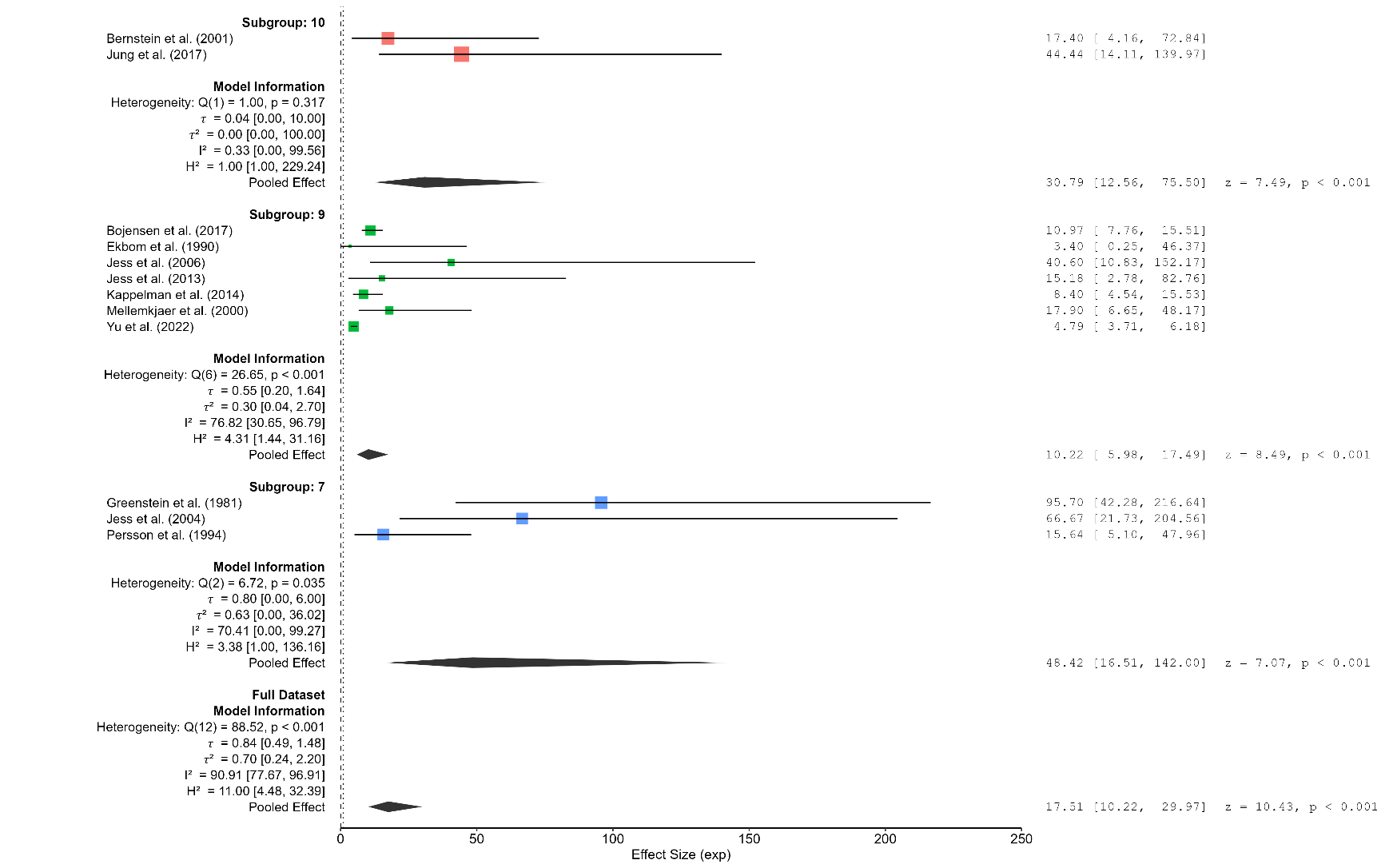


**Supplementary Figure 19.** SIR SBC with 95% C.I. cancer in Crohn’s Disease (log scale) in separated estimation for ICD at the beginning of the study. Individual and combined standardized incidence ratios (with 95% confidence intervals) of colorectal cancer in Crohn’s Disease. The size of the boxes is proportional to the weight (1/SE) of each study.


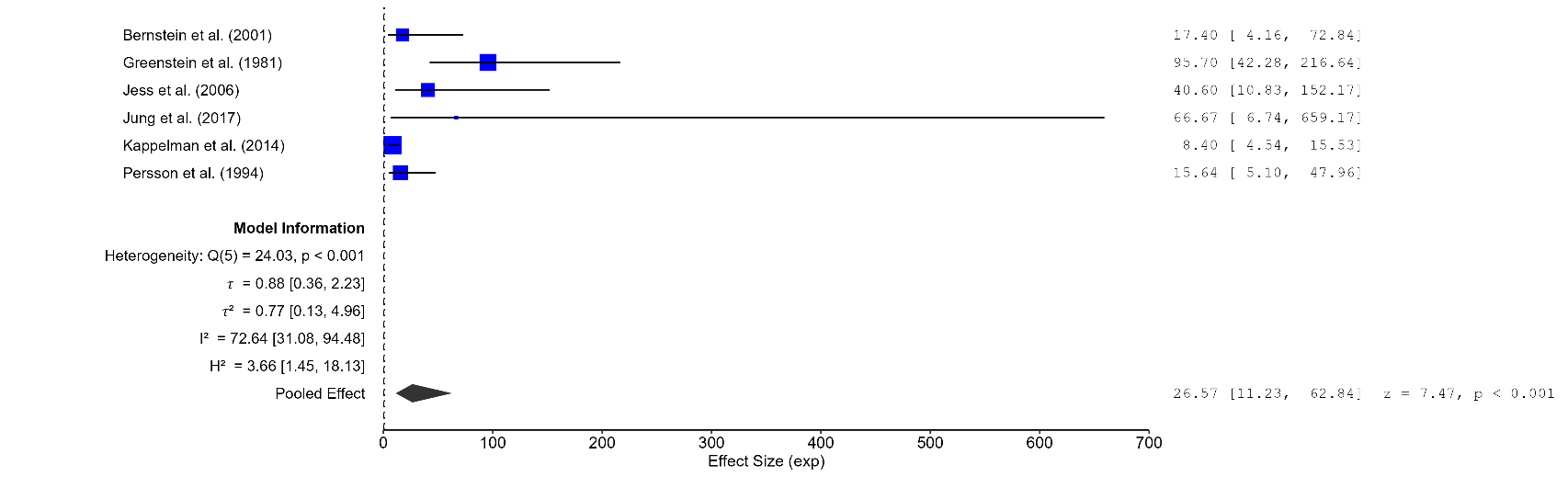


**Supplementary Figure 20.** SIR SBC with 95% C.I. cancer in Crohn’s Disease (log scale) in separated estimation for each nation by follow-up. Individual and combined standardized incidence ratios (with 95% confidence intervals) of colorectal cancer in Crohn’s Disease. The size of the boxes is proportional to the weight (1/SE) of each study.


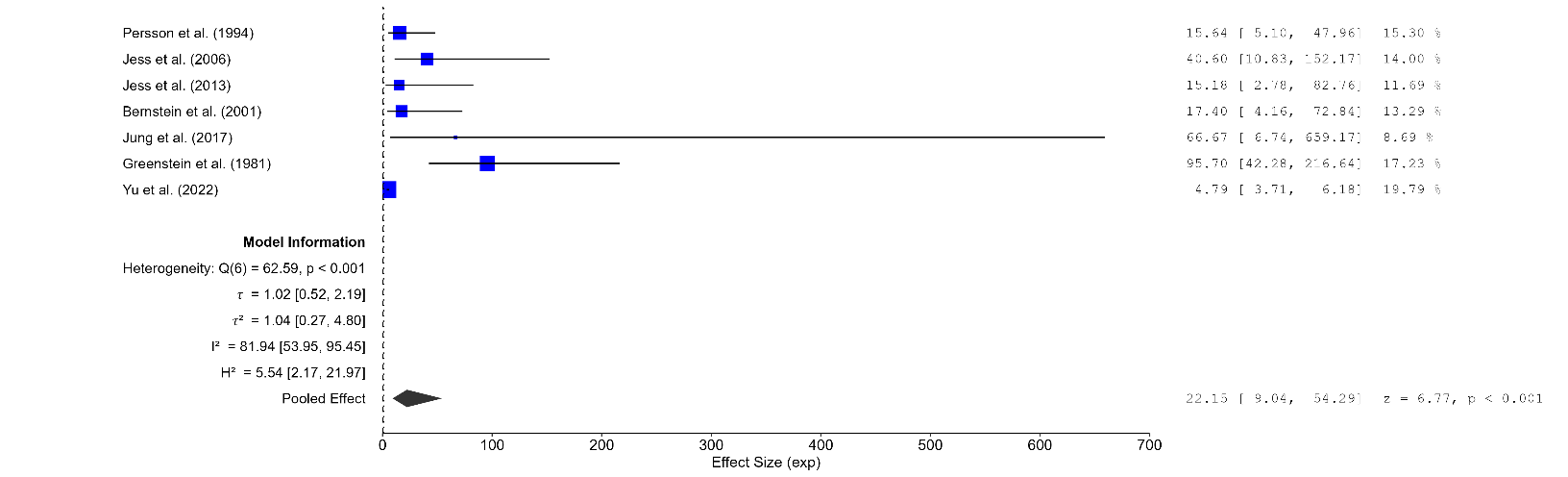


**Supplementary Figure 21.** SIR SBC with 95% C.I. cancer in Crohn’s Disease (log scale) in separated estimation for each nation by major cases. Individual and combined standardized incidence ratios (with 95% confidence intervals) of colorectal cancer in Crohn’s Disease. The size of the boxes is proportional to the weight (1/SE) of each study.


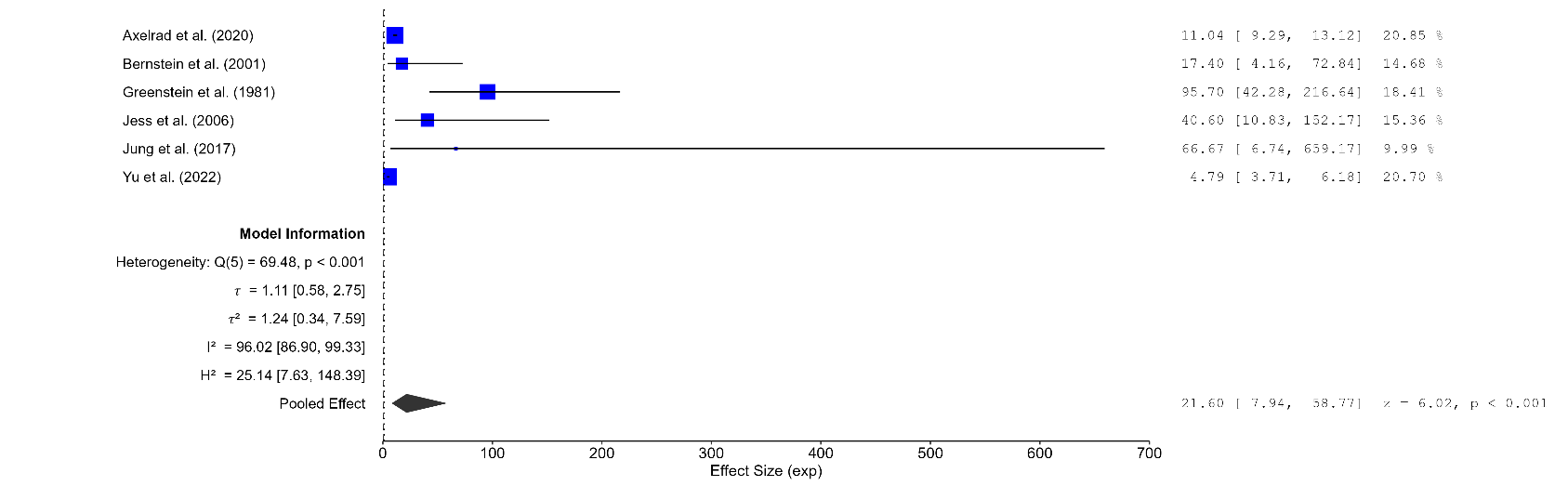


**Supplementary Figure 22.** SIR SBC with 95% C.I. cancer in Crohn’s Disease (log scale) in separated estimation for each nation by major population. Individual and combined standardized incidence ratios (with 95% confidence intervals) of colorectal cancer in Crohn’s Disease. The size of the boxes is proportional to the weight (1/SE) of each study.


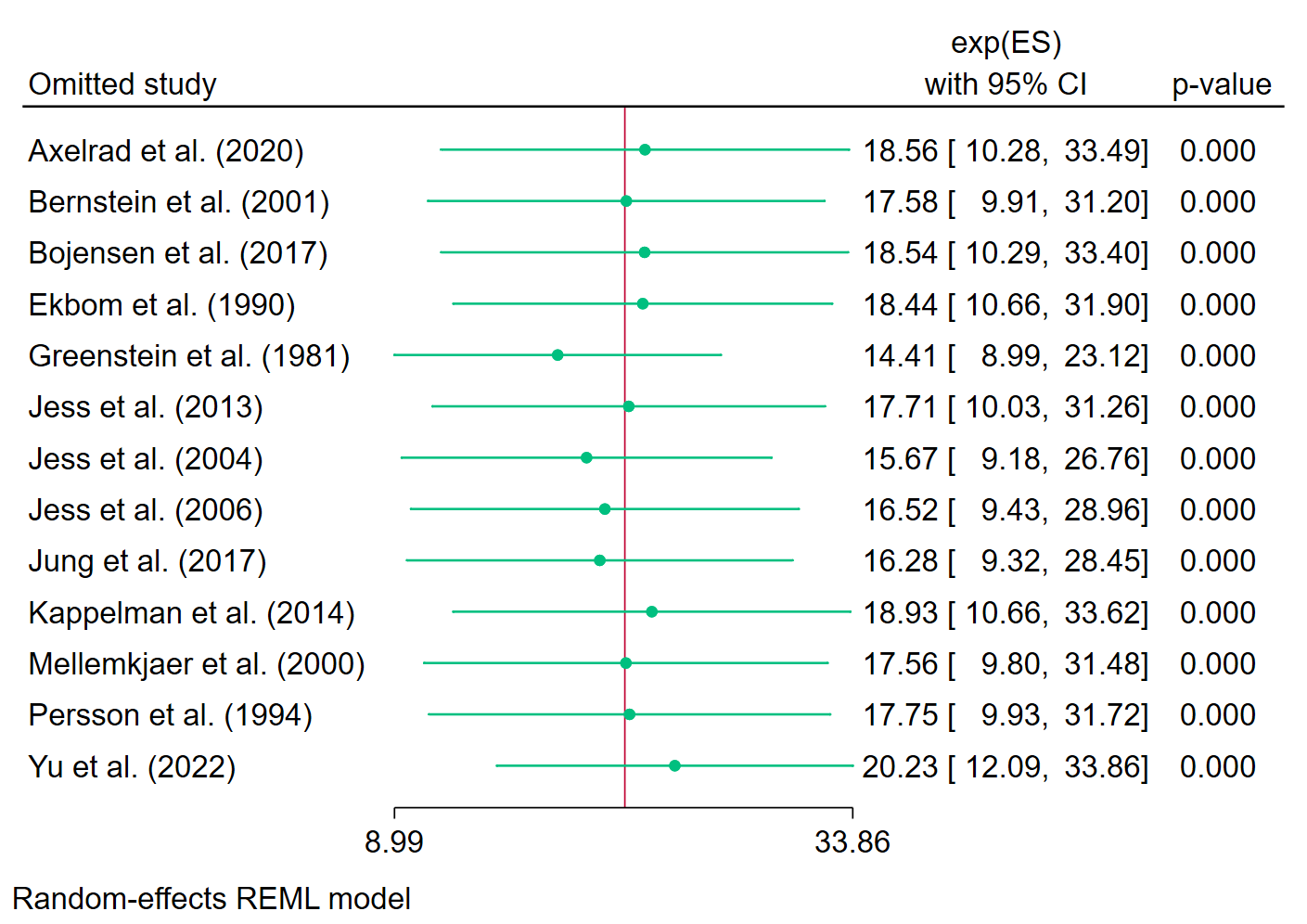


**Supplementary Figure 23.** Leave-one-out sensitivity analysis of the meta-analysis in SBC. For each included study, the pooled effect estimate (exp(ES)) and its Confidence Interval at 95% are reported. Effect estimates were obtained using a random-effects model fitted by restricted maximum likelihood (REML) with Knapp–Hartung adjustment for standard errors.


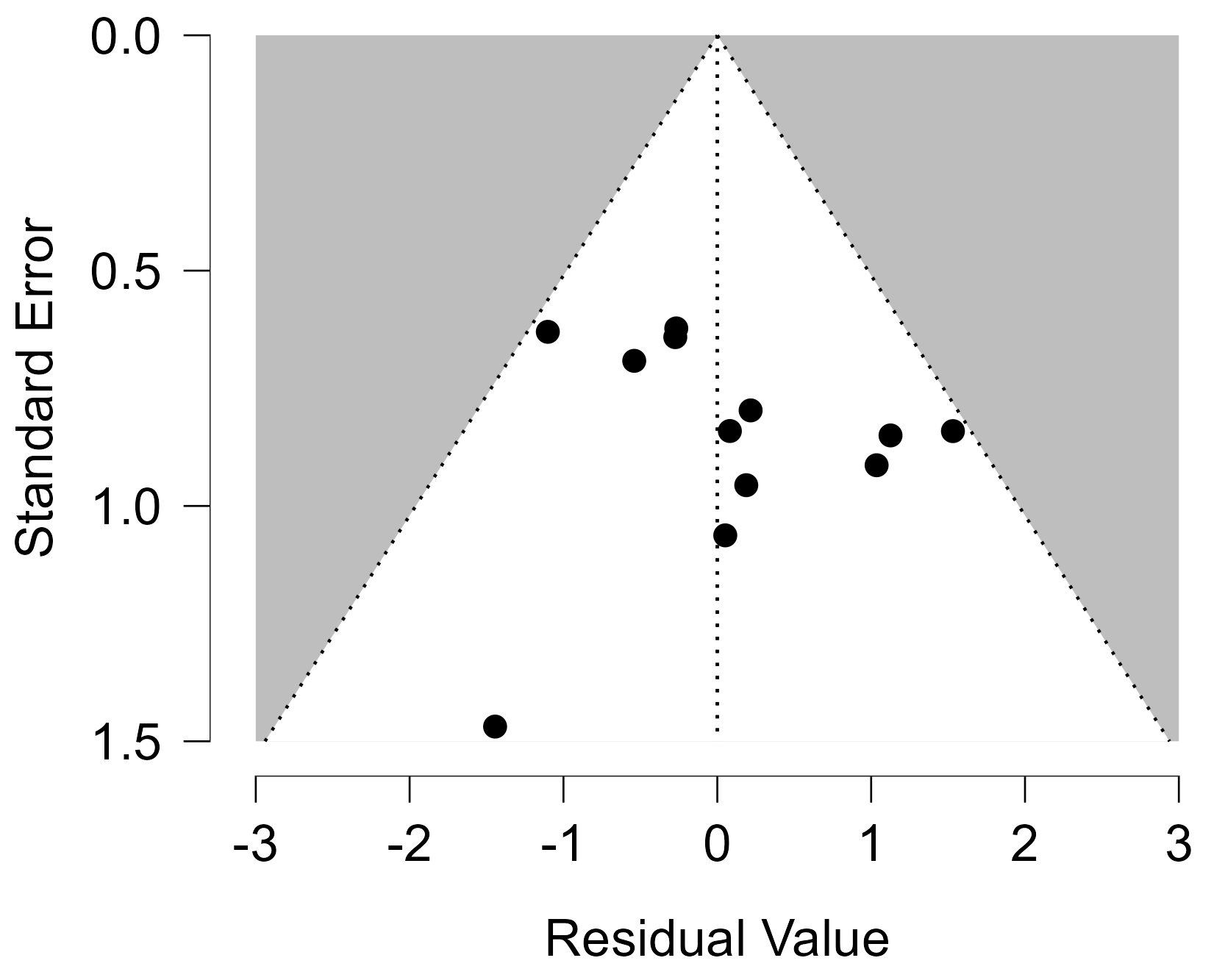


**Supplementary Figure 24.** Funnel plot SBC. Each dot represents a single study, plotted with its effect size on the x-axis and its standard error on the y-axis. The vertical red line represents the overall effect estimate derived from the random-effects model using restricted maximum likelihood (REML). The pseudo 95% confidence interval region, shown as gray diagonal lines, outlines the expected spread of studies in the absence of bias or between-study heterogeneity.


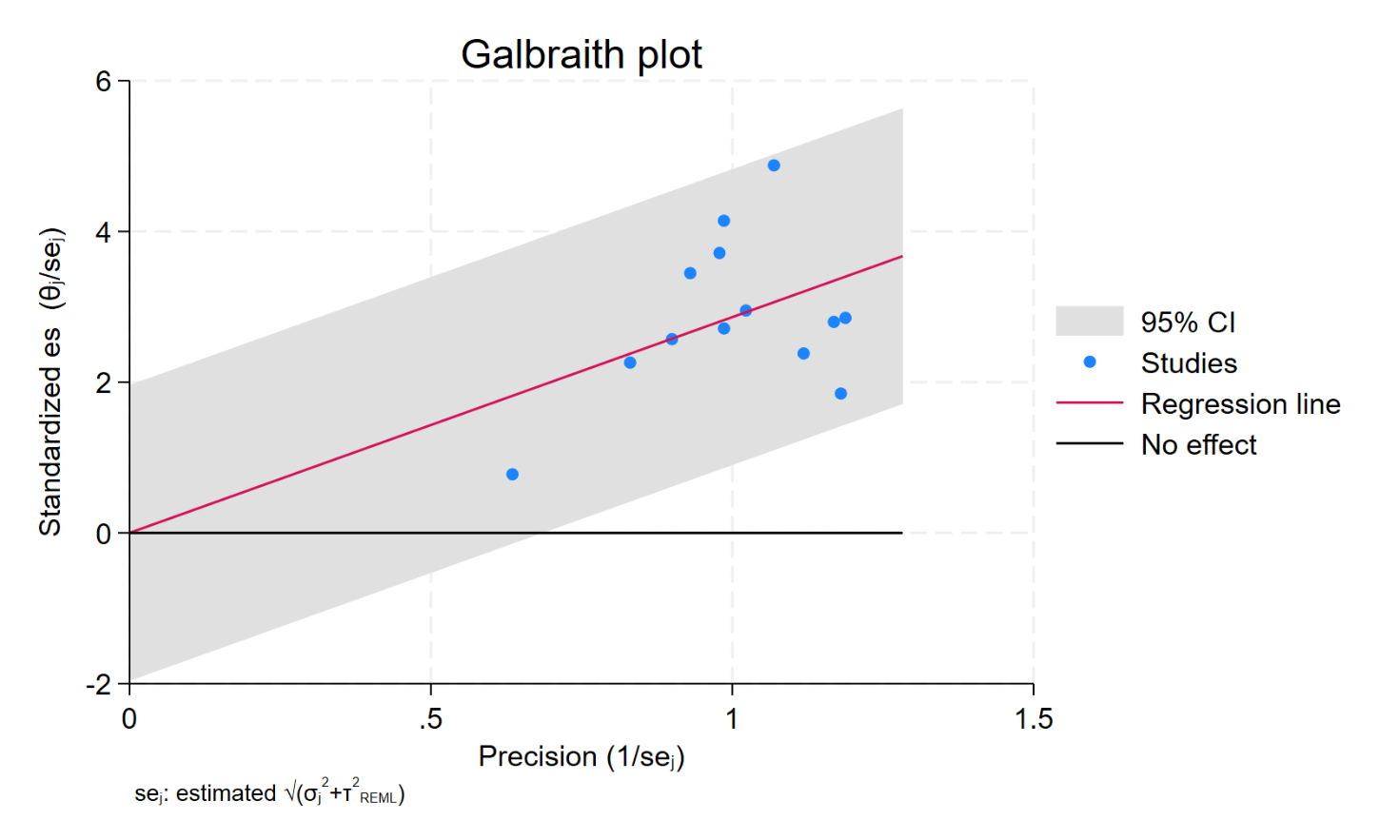


**Supplementary Figure 25.** Galbraith plot SBC. Each study is represented as a point, where the x-axis denotes the precision (defined as the inverse of the standard error), and the y-axis indicates the standardized effect size. The red regression line represents the overall summary effect estimated from the random-effects model, and the grey shaded area corresponds to the confidence interval at 95% around regression line. The horizontal black line at y = 0 corresponds to the line of no effect, serving as a reference.


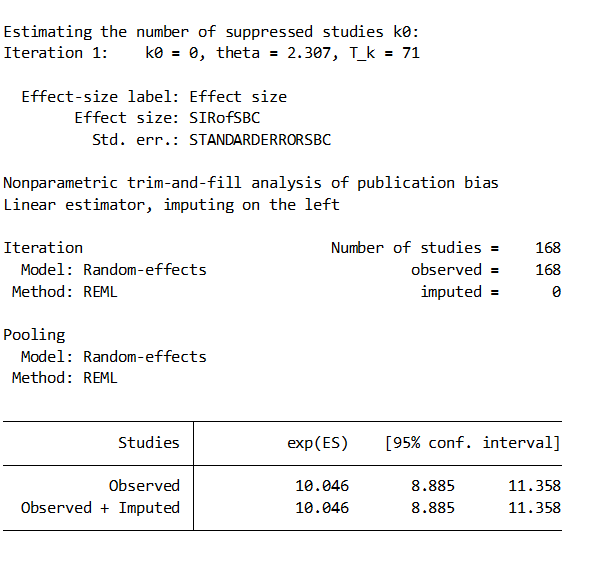


**Supplementary Figure 26.** Trim-and-fill analysis for assessment of publication bias SBC. A nonparametric trim-and-fill procedure was applied using a random-effects model estimated by restricted maximum likelihood (REML) to evaluate the potential impact of publication bias.


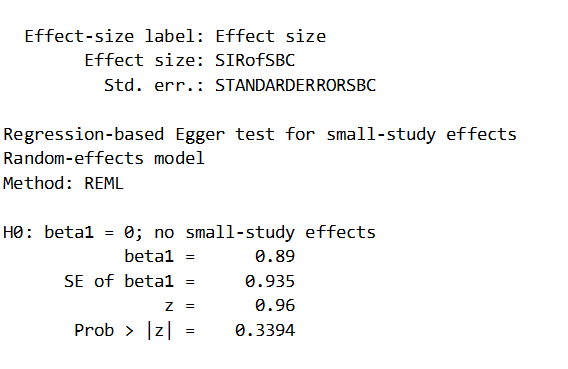


**Supplementary Figure 27.** Egger’s test for small-study effects in a meta-analysis in SBC. A random-effects model was fitted using the REML method.


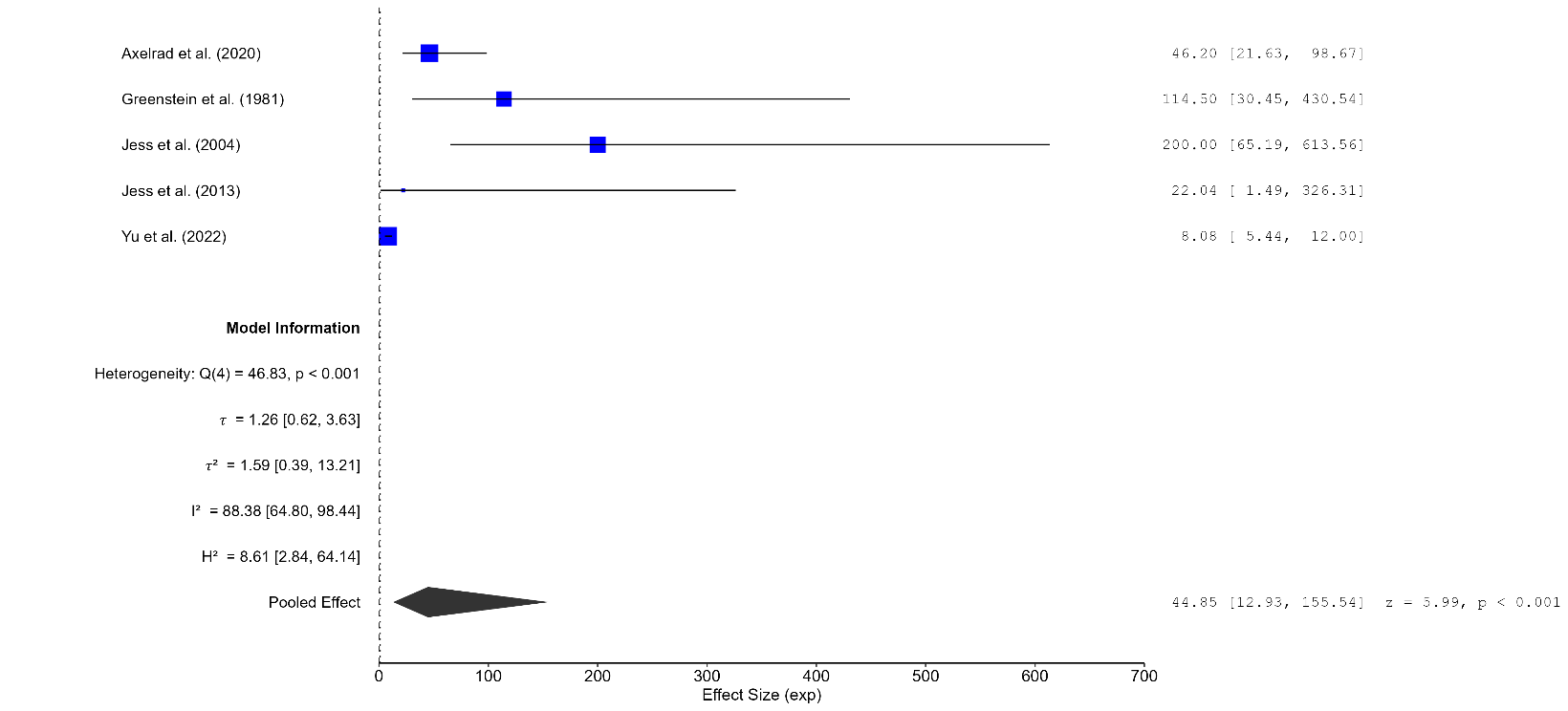


**Supplementary Figure 28.** SIR SBC with 95% C.I. for L1 cancer in Crohn’s Disease (log scale) in separated estimation. Individual and combined standardized incidence ratios (with 95% confidence intervals) of colorectal cancer in Crohn’s Disease. The size of the boxes is proportional to the weight (1/SE) of each study.


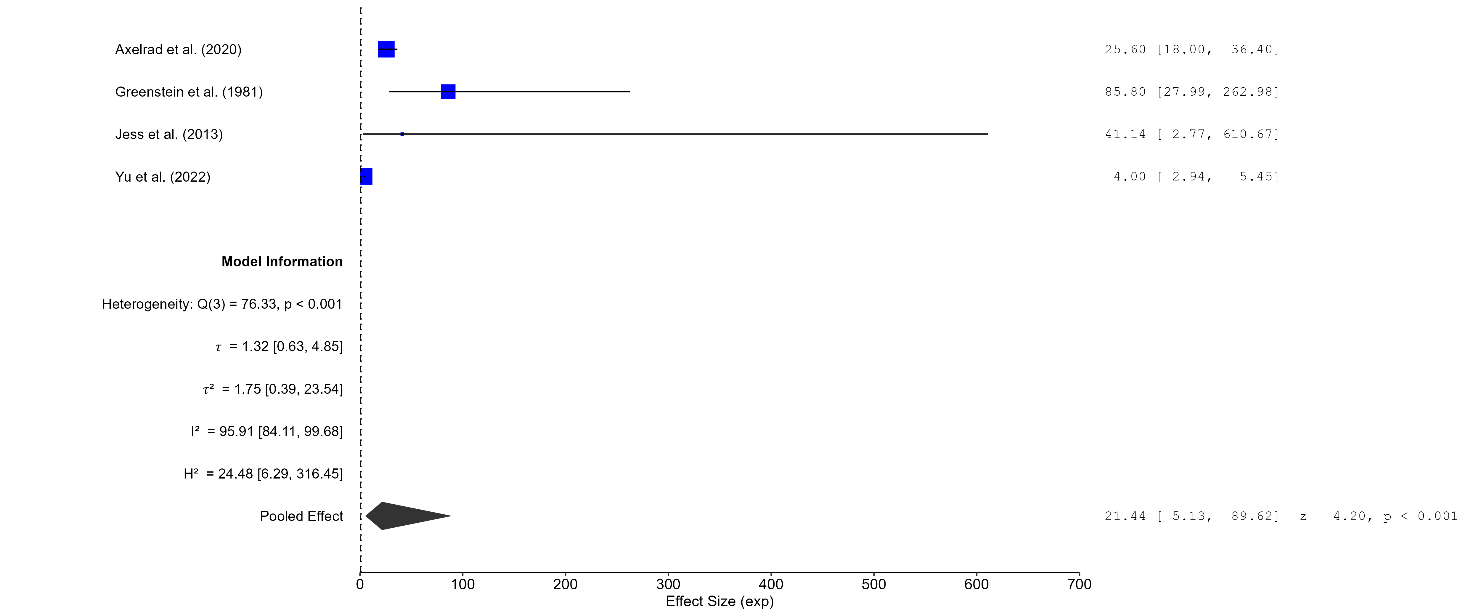


**Supplementary Figure 29.** SIR SBC with 95% C.I. for L3 cancer in Crohn’s Disease (log scale) in separated estimation. Individual and combined standardized incidence ratios (with 95% confidence intervals) of colorectal cancer in Crohn’s Disease. The size of the boxes is proportional to the weight (1/SE) of each study.
